# Retrieval-Augmented Large Language Model Counseling for Continuous Glucose Monitoring in Diabetes: Source-Masked Multirater Comparative Evaluation

**DOI:** 10.2196/98519

**Published:** 2026-07-31

**Authors:** Zhijun Guo, Alvina Lai, Emmanouil Korakas, Aristeidis Vagenas, Irshad Ahamed, Christo Albor, Hengrui Zhang, Justin Healy, Kezhi Li

**Affiliations:** 1 Institute of Health Informatics, University College London, 222 Euston Road, London, NW1 2DA, United Kingdom, 44 7859995590; 2 University College London Hospitals NHS Foundation Trust, London, United Kingdom, London, United Kingdom; 3 Dartford and Gravesham NHS Foundation Trust, Dartford, United Kingdom, Dartford, England, United Kingdom; 4 Royal Free London NHS Foundation Trust, London, United Kingdom

**Keywords:** continuous glucose monitoring, large language model, retrieval augmented generation, conversational agent, patient-facing AI, clinical evaluation, diabetes care

## Abstract

**Background:**

Continuous glucose monitoring (CGM) is central to diabetes care, but explaining CGM patterns consistently and empathetically remains time-intensive in clinical practice. Large language model (LLM)–based systems may support patient-facing interpretation of CGM data, but evidence remains limited for retrieval-grounded tools evaluated against clinician-authored responses in counseling scenarios. The system was intended for CGM interpretation and communication support rather than autonomous therapeutic decision-making.

**Objective:**

This study aimed to evaluate whether a retrieval-grounded LLM-based conversational agent (CA) could support patient understanding of CGM data and preparation for diabetes consultations by generating responses to questions arising during CGM-informed diabetes counseling, with quality comparable to clinician-authored responses.

**Methods:**

We developed a scaffolded LLM-based CA for CGM interpretation and diabetes counseling support. The system was designed to provide plain-language explanations of CGM patterns and responses to diabetes management questions while avoiding directive or individualized medical advice, such as recommending medication initiation, dose adjustment, or regimen changes. Around 12 CGM-informed cases, each comprising a deidentified CGM trace, a synthetic patient vignette, and accompanying CGM visual materials, were constructed from using available clinical datasets. Between October 2025 and February 2026, 6 senior UK diabetes clinicians each reviewed 2 assigned cases and answered 24 questions (12 per case). In a source-masked multirater evaluation, each CA-generated and clinician-authored response was independently rated by 3 clinicians on 6 quality dimensions, including clinical accuracy, guideline adherence, actionability, personalization, communication clarity, and empathy. Safety flags and perceived source labels were also recorded. The primary analysis used linear mixed effects models with random intercepts for case and rater.

**Results:**

A total of 288 unique responses (144 CA and 144 clinician responses) were evaluated, generating 864 ratings. CA-generated responses received higher quality scores than clinician-authored responses under controlled vignette-based conditions, with mean scores of 4.37 (SD 0.57) versus 3.58 (SD 0.90) and an estimated mean difference of 0.782 points on a 5-point scale (95% CI 0.692‐0.872; *P*<.001). This pattern was observed across all 6 categories of patient questions. The largest estimated differences were for empathy (mean difference 1.062, 95% CI 0.948‐1.177) and actionability (0.992, 95% CI 0.877‐1.106). Safety flag distributions were similar between CA and clinician responses, with major concerns rare in both groups (n=3, 0.7% each). Although CA responses were longer, additional analyses adjusting for word count did not indicate that response length explained the overall quality difference.

**Conclusions:**

Scaffolded LLM-based systems may have value as adjunct tools for CGM review, patient education, and preconsultation preparation by supporting standardized explanatory tasks. However, these findings should be interpreted in light of the vignette-based design, restricted datasets, and a small clinician panel, and they do not establish suitability for autonomous therapeutic decision-making, medication adjustment, or unsupervised real-world use. Prospective validation in clinical workflows is needed before implementation.

## Introduction

### Background

Diabetes mellitus is a chronic metabolic disease characterized by hyperglycemia due to impaired insulin secretion, insulin action, or both, and is associated with substantial microvascular and macrovascular complications [1]. The International Diabetes Federation (IDF) reports that in 2024, approximately 589 million adults aged 20‐79 years were living with diabetes worldwide (11.1% of the adult population), with this number projected to reach 853 million by 2050, an increase of 46% [2]. Given this growing global burden, digital tools are increasingly being investigated to support day-to-day monitoring, interpretation of glycemic patterns, and communication around self-management [3,4].

Continuous glucose monitoring (CGM) has become central to contemporary diabetes care [5]. CGM systems provide near real-time interstitial glucose readings and trend information, enabling assessment of time in range (TIR), glycemic variability, and hypoglycemia risk [6,7]. Evidence from clinical trials and consensus reports indicates that CGM use can increase TIR, reduce hypoglycemia, and improve treatment satisfaction in both type 1 and type 2 diabetes [6,8]. Recent American Diabetes Association (ADA) Standards of Care recommend CGM for most individuals on intensive insulin therapy and support broader adoption where feasible [1,9]. As device accuracy, wearability, and reimbursement have improved, CGM use in routine clinical practice has expanded rapidly [9,10].

Despite these benefits, interpreting CGM data remains challenging for many people living with diabetes. Although uptake is increasing, access and sustained use remain affected by device cost, insurance coverage, and variation in prescribing practices across clinical settings [11]. Modern CGM platforms increasingly provide structured summaries of glucose data for patient and clinical review, including pattern reports, trend visualizations, and summary statistics [12,13]. Dexcom Clarity, for example, highlights glucose patterns, trends, and statistics through report-based summaries [12], whereas Abbott’s LibreView provides reports such as Glucose Pattern Insights that highlight glycemic patterns and may include medication and lifestyle considerations for review [13]. However, these tools are primarily designed to summarize and visualize data rather than to provide conversational, patient-facing explanations of why patterns may be occurring or how they relate to daily behaviors, treatment routines, and patient concerns. Even among CGM users, limited structured training in how to interpret traces means that many still feel overwhelmed by the volume and complexity of CGM readouts and struggle to relate observed patterns to food intake, physical activity, medication timing, or other day-to-day behaviors [14]. Patients may be unsure how to respond to recurring highs or lows, how to understand trends over time, or which questions should be brought to clinicians during review [15-17]. Limited consultation time further constrains opportunities for detailed, individualized explanation of CGM traces in standard clinical encounters [14,15]. Together, these practical and educational barriers highlight a need for scalable approaches that can support clear, timely, and patient-facing interpretation of CGM data alongside routine care.

In addition to these interpretive challenges, many people living with diabetes experience substantial emotional burden. Meta-analyses suggest that approximately 10%‐15% of adults with diabetes have clinically significant depressive symptoms [18]. Diabetes distress and depression are associated with poorer glycemic control, reduced adherence to self-management behaviors, and lower quality of life [19]. In this context, support tools for CGM-related communication must address not only the technical explanation of glucose patterns but also the uncertainty, frustration, and anxiety that often accompany diabetes self-management. This does not necessarily require formal psychological intervention, but it does underscore the importance of clarity, reassurance, and empathic communication in patient-facing diabetes support.

Recent advances in large language models (LLMs) offer a potential way to address unmet informational and communication needs in diabetes care [20]. LLMs can accept structured and unstructured inputs, including numerical summaries, clinical text, and free-text patient queries, and generate contextualized natural-language explanations [21]. This makes them candidates for supporting tasks such as CGM interpretation, patient education, and communication of case-specific information in accessible language [22,23]. Early evaluations and review articles report that, in many scenarios, LLM-based systems provide reasonably accurate health information and can generate responses that show elements of cognitive empathy, such as recognizing expressed emotions and offering supportive language in simulated patient interactions [22-25]. However, current LLMs in medicine remain limited by hallucinated or incomplete content, variable alignment with clinical guidelines, and challenges in transparently incorporating patient-specific structured data, which raises concerns about their safe use in clinical contexts [22,26]. Retrieval-augmented generation (RAG) has been proposed as one strategy to mitigate these limitations by grounding LLM outputs in curated, evidence-based sources and explicitly linking responses to both guideline documents and case-specific data such as CGM traces [25,26]. Empirical evidence on the performance of such systems in patient-specific, CGM-informed diabetes scenarios, however, remains limited.

Recent studies have applied LLM- or RAG-based conversational agents (CAs) to diabetes care, mainly for diabetes education, lifestyle, and self-management advice, or general question and answering [27,28]. These systems are commonly evaluated in terms of diabetes knowledge, perceived usefulness, usability, and the appropriateness or safety of chatbot advice [27-30], but they rarely incorporate CGM data directly or examine how such systems perform when asked to interpret CGM-informed cases in a patient-facing manner. In parallel, other studies have used LLMs to analyze CGM data by calculating standard CGM metrics and generating narrative summaries of glucose traces, which endocrinologists then assess for accuracy, completeness, and safety, often using simulator-generated rather than real-world data [31]. Collectively, these studies suggest that LLM-based tools may assist with diabetes-related information provision and CGM analysis [27-31]. However, they have not adequately evaluated retrieval-grounded LLM-based CAs in CGM-informed scenarios that require structured glucose interpretation together with patient-centered communication, nor have they compared specialist ratings of CA-generated responses against clinician-authored responses to the same cases.

In practice, one plausible role for such a system may be as an adjunct to routine diabetes care, particularly for preconsultation preparation, standardized explanation of CGM patterns, and support for common patient-facing questions that are structured but time-consuming to address repeatedly in routine care [32,33]. Before a scheduled review, patients might use such a system to obtain a plain-language explanation of recent glucose patterns, identify recurring concerns, and formulate questions for discussion with their diabetes care team. In this way, the system could potentially help surface common issues in advance, support more consistent handling of routine CGM-related questions, and improve consultation efficiency by allowing clinicians to focus more directly on individualized decision-making and more complex cases. More broadly, this suggests a possible practical role for LLM-based tools in diabetes services, where many routine advisory tasks involve synthesizing structured and semistructured information into clear, context-specific communication, while final treatment recommendations and higher-risk clinical judgments remain clinician-led. Accordingly, the potential value of such systems may lie less in autonomous decision-making than in supporting routine explanatory work, augmenting specialist capacity, and helping prioritize clinician time toward situations requiring greater clinical complexity or safety oversight [33,34].

### Study Aim

To our knowledge, this study is among the first to conduct a source-masked comparative evaluation in which diabetes specialists assessed both retrieval-grounded LLM-generated and clinician-authored responses to identical CGM-informed cases across predefined clinical and psychosocial evaluation criteria. To address the need for scalable support in patient-facing communication around CGM review, we developed a scaffolded LLM GPT-5.1–based LLM CA incorporating RAG that generated structured, plain-language responses to questions arising in CGM-informed diabetes counseling using case-specific CGM information, vignette context, and guideline-based retrieval. We then conducted a source-masked multirater evaluation between October 2025 and February 2026 with UK diabetes clinicians, who assessed CA- and clinician-authored responses for quality and safety. In doing so, this study examined whether such a system could support standardized explanatory and educational aspects of diabetes care in consultations involving CGM review, without positioning it as a substitute for clinician judgment.

## Methods

### Ethical Considerations

This study did not involve direct contact with patients or access to identifiable clinical records. The CGM files were derived from publicly available, preexisting, deidentified clinical datasets, and all accompanying patient vignettes were synthetic and created solely for research purposes. The human contributors were 6 diabetes clinicians who served as expert reviewers by providing written responses and rating anonymized text outputs. They were not asked to provide personal or sensitive information and were considered expert assessors rather than research participants. According to UK Health Research Authority guidance, research using publicly available or fully anonymized data in which individuals cannot be identified may not require formal Research Ethics Committee review [35]. Because this study used publicly available anonymized CGM datasets together with clinician ratings of generated responses and did not involve identifiable patient information, formal ethics approval was not required.

### CGM Data and Case Vignettes

**Figure 1.**
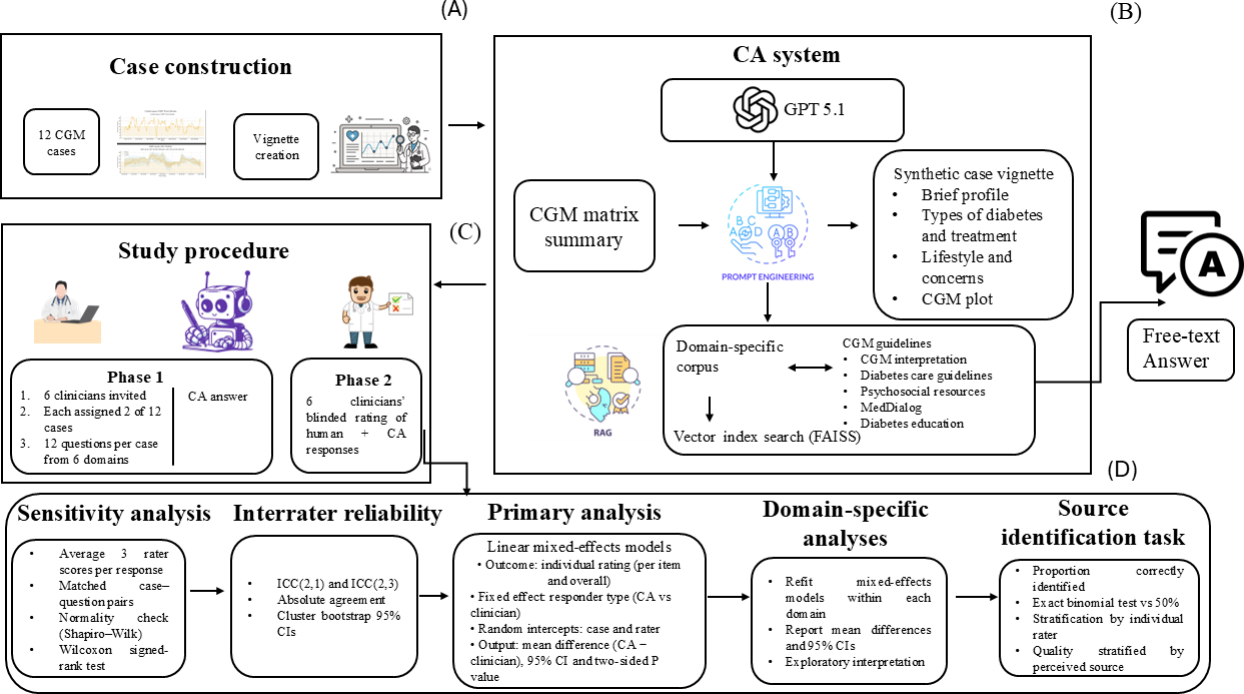
Overview of the study workflow. (**A**) Construction of 12 deidentified continuous glucose monitoring (CGM) cases and corresponding synthetic patient vignettes. (**B**) Retrieval-grounded conversational agent (CA) system using GPT-5.1, structured CGM summaries, vignette context, and guideline excerpts retrieved through retrieval-augmented generation (RAG). (**C**) Two-phase clinician study involving response generation and source-masked rating. (**D**) Statistical analyses, including interrater reliability assessment, mixed effects models, sensitivity analyses, domain-specific analyses, and source-identification analyses. CA: conversational agent; CGM: Continuous glucose monitoring; FAISS: Facebook AI similarity search; ICC: intraclass correlation coefficient.

An overview of the complete workflow is presented in Figures 1A-D. We constructed 12 CGM-based cases using deidentified glucose traces from 2 publicly available research datasets. Six traces (3 type 1 diabetes and 3 type 2 diabetes) were sampled from the ShanghaiT1DM and ShanghaiT2DM datasets [36], which provide 3‐14 days of CGM values at 15-minute intervals for 12 individuals with type 1 diabetes and 100 individuals with type 2 diabetes, together with capillary blood glucose measurements, blood ketone, self-reported dietary intake, insulin doses, and clinical characteristics [36]. For the 6 Shanghai cases used in this study, the CGM monitoring period ranged from 7 to 14 days: 1 case included 7 consecutive days of data, and the remaining 5 included at least 10 days of CGM readings. In addition to the CGM glucose time series, selected contextual fields from the source datasets, including dietary intake and insulin records, were used to inform the construction of the synthetic patient vignettes. These variables were used only for case contextualization and were not analyzed as separate study variables.

The remaining 6 traces were selected from the OhioT1DM 2018 dataset [37], which contains 8 weeks of CGM, insulin pump, physiological sensor, and self-reported life-event data for 6 adults with type 1 diabetes [37]. In addition to the CGM glucose measurements, selected self-reported life event information was used to inform the construction of the synthetic patient vignettes. As with the Shanghai cases, these variables were used only for case contextualization and were not analyzed as separate study variables. The resulting case set comprised 9 type 1 diabetes cases and 3 type 2 diabetes cases. This distribution reflected both the composition of the source datasets and the more limited availability of suitable publicly available type 2 diabetes CGM datasets for case construction, while allowing the evaluation to include both major diabetes types and a range of CGM pattern presentations. Within the type 2 diabetes group, cases were not further selected to represent specific treatment regimens, such as insulin-treated or noninsulin-treated subgroups. Instead, the selected cases should be interpreted as examples of CGM-informed counseling scenarios in type 2 diabetes rather than as representing the full clinical heterogeneity of the broader type 2 diabetes population.

To approximate a realistic consultation context while preserving privacy, we created a synthetic case vignette for each selected CGM trace. Each vignette included a brief clinical profile, such as age band, sex, diabetes type and duration, treatment regimen, typical diet and activity patterns, and the patient’s main concerns about glycemic control, together with one or more CGM plots (an example can be found in Multimedia Appendix 1) generated from the underlying trace to provide clinicians with an intuitive visual overview of glucose patterns. For both datasets, the CGM glucose traces provided the primary basis for case selection, CGM metric calculation, and visual material generation, whereas available contextual fields, including dietary intake, insulin records, treatment information, clinical characteristics, and self-reported life-event details, were used only as anchors for constructing synthetic vignettes and were not analyzed as separate study variables. Where such fields were incomplete, details were synthetically expanded only to provide plausible counseling context. Coherence was assessed by checking consistency with diabetes type, treatment modality, CGM summary metrics, and visible glucose patterns, including TIR, time above range (TAR), time below range (TBR), glycemic variability, nocturnal lows, postprandial excursions, fasting or early-morning rises, and data completeness. Draft vignettes and plots were prepared by ZG and refined through formative review with senior diabetologist EK; refinements were limited to improving clinical plausibility, differentiating type 1 and type 2 diabetes contexts, and representing early-morning glucose rise or possible dawn phenomenon where supported by the CGM trace.

### CA Design

A retrieval-grounded CA for CGM interpretation and diabetes-related counseling scenarios was implemented using the GPT-5.1 LLM accessed via the OpenAI API. GPT-5.1 was one of the most advanced models available at the time of system development; no comparative benchmarking across alternative models was undertaken. The base model was used without parameter fine-tuning. Task adaptation relied on structured prompting combined with RAG. Deidentified CGM summaries and vignette-based patient queries were embedded into predefined prompt templates, and guideline-aligned reference materials were retrieved from a curated knowledge base to condition response generation.

For each case, the CGM glucose time series was processed in Python using a custom analysis function that calculated core consensus metrics, including monitoring duration, data completeness, mean glucose, SD, coefficient of variation, and the percentage of readings within standard CGM ranges (TIR 70‐180 mg/dL [3.9‐10.0 mmol/L], TBR <70 mg/dL [<3.9 mmol/L] and <54 mg/dL [<3.0 mmol/L], and TAR >180 mg/dL [>10.0 mmol/L] and >250 mg/dL [>13.9 mmol/L]), consistent with international recommendations for CGM-derived metrics and TIR reporting [6]. The function generated a concise text summary listing these metrics alongside commonly used target thresholds. This standardized CGM summary was used as structured input to the CA.

To verify the correctness of the implementation, ZG manually recalculated the CGM metrics for representative traces from both the Shanghai and Ohio datasets using Microsoft Excel as an implementation check. For these validation traces, all metrics were recomputed using simple count and average formulas based on the international CGM TIR consensus definitions [38], and the Excel results exactly matched the Python (Python Software Foundation) outputs at the precision reported. This validation step was performed solely to confirm the correctness of the implementation and to support basic quality checks, such as visual inspection, so that the reported metrics were consistent with the plotted CGM traces. The metric calculations themselves were not shown to clinician raters. For each question, the model input consisted of the CGM summary, the synthetic case vignette (clinical profile and contextual information), the text of a prespecified question from the case-specific question set, and image-based CGM visualizations for the case, including both the continuous glucose trace and an ambulatory glucose profile (AGP)-style profile. These visualizations were supplied directly to the CA as multimodal inputs, so the model’s interpretation was informed by both structured summary metrics and visual glucose pattern information. An example CGM summary format is provided in Multimedia Appendix 1.

To ground responses in current evidence and professional guidance, a RAG approach was used. A domain-specific corpus was built from publicly available materials on CGM metrics and interpretation [38-41], CGM-guided glucose management [42-46], AGP reports [39], major diabetes care guidelines [47,48], and resources on emotional and psychosocial aspects of diabetes [47-51]. The corpus also included selected segments from the MedDialog 2020 medical dialogue dataset to provide examples of clinician-patient conversational style [52] and curated patient education materials from national health services and diabetes charities (such as Diabetes UK [50] and the ADA [51]), which were manually organized into topic-based summaries. All documents were converted to plain text and split into short overlapping segments using a recursive character-based splitter with a target segment length of approximately 500 characters and an overlap of 100 characters. Each segment was embedded using OpenAI’s text embedding service via the LangChain OpenAIEmbeddings wrapper [53] and stored in a Facebook AI Similarity Search (FAISS) vector index implementing approximate nearest-neighbor search [54]. At inference time, the current question text was used as the retrieval query to obtain the top 2 nearest segments from the FAISS index. The retrieval function returned a Euclidean (L2) distance score, with lower values indicating closer embedding-space proximity.

Two prompt templates were used to structure model behavior across interaction stages. The first template was used to generate an initial plain-language explanation of the precomputed CGM summary and vignette context, with retrieved guideline-aligned reference materials incorporated through the RAG pipeline to support interpretation. The CGM metrics themselves were computed in Python in advance and provided to the model as structured input rather than being generated by the prompt. The second template was used to answer follow-up patient questions, again incorporating retrieved reference materials through the RAG pipeline to support context-aware responses. Full prompt templates are provided in Multimedia Appendix 2. The system prompt defined the CA as a diabetes specialist and instructed it to provide clear, plain-language, empathetic, and nonjudgmental explanations aligned with standard diabetes education practices. During system development, selected example outputs were reviewed by a clinical collaborator (EK) to provide targeted feedback on tone, clarity, and clinical appropriateness, and this feedback informed the final prompt refinement. For each case, question pair in the formal evaluation, the model produced a single free-text response without iterative refinement.

For formal evaluation, all CA responses were generated on November 14, 2025, using the OpenAI API model identifier gpt-5.1. A fixed configuration was applied to all formal case-question pairs. Generation parameters were set to a temperature of 0.1, a top_p value=1.0, and maximum output length of 1200 tokens. Frequency and presence penalties were left at default settings, no stop sequences were specified, and no seed parameter was specified in the API call. The low temperature setting was selected to reduce output variability and limit speculative generation [22]. API credentials were stored as environment variables and were not embedded in the code or shared materials. For retrieval, documents were embedded using text-embedding-3-small, split into approximately 500-character chunks with 100-character overlap, indexed in FAISS, and retrieved with top_k=2 using the current question text as the retrieval query.

### Safety and Security

Several safeguards were implemented to minimize the risk of inappropriate, misleading, or clinically unsafe outputs. Inputs to the CA were restricted to deidentified CGM summaries and synthetic vignettes, so no identifiable patient information or directly actionable treatment instructions were supplied. Behavioral constraints embedded in the system prompted the CA to avoid prescriptive therapeutic recommendations and to encourage consultation with the person’s usual diabetes care team when glycemic patterns suggested potential risk, reinforcing its role as a communication support tool for structured CGM explanation rather than a substitute for clinical judgment. The RAG component was designed to expose the model to a curated, guideline-consistent corpus rather than unrestricted external sources, supporting retrieval traceability and potential guideline alignment; all CGM metrics used to contextualize model reasoning were validated in advance to avoid propagating incorrect numerical information. During system development, draft outputs, for example, case-question pairs, were iteratively reviewed by diabetes clinicians, and their feedback was used to refine the prompt and guardrails until the response style was judged acceptable for the study. Potential failure modes considered during system design included misinterpretation of CGM patterns, overgeneralized lifestyle advice, excessive reassurance, inappropriate specificity in medication-related responses, failure to recognize clinically concerning hypoglycemia or hyperglycemia patterns, and mismatch with local prescribing or eligibility criteria. These risks were mitigated through prompt-based safety constraints, retrieval from a curated guideline-consistent corpus, clinician review during development, and the use of safety flags in the formal evaluation.

### Study Design

This study used a vignette-based, multirater, source-masked comparative design to evaluate the CA’s responses against those of diabetes clinicians. The expert panel comprised 6 senior diabetes specialists recruited from major UK National Health Service (NHS) Foundation Trusts, including Imperial College Healthcare NHS Trust (St Mary’s Hospital), University College London Hospitals NHS Foundation Trust (UCLH), and the Royal Free London NHS Foundation Trust. The use of senior diabetes specialists provided a stringent benchmark for comparison.

Participants met predefined eligibility criteria: (1) a primary clinical appointment within a tertiary referral center or major teaching hospital; (2) at least 10 years of postqualification clinical experience; and (3) demonstrated expertise in diabetes technology, particularly in the clinical interpretation of CGM and advanced insulin delivery systems. The panel included consultant diabetologists and senior specialists, several of whom hold clinical-academic appointments.

After constructing 12 CGM-based cases with synthetic clinical vignettes as described above, 6 UK diabetes clinicians were first invited to provide written responses and subsequently to rate anonymized response pairs. Each clinician was randomly assigned 2 of the 12 cases and received the full standardized case package for each assigned case. This included the synthetic vignette together with structured supporting materials, including demographic and clinical background, current treatment, CGM summary metrics, lifestyle and self-management information, patient-reported observations, accompanying CGM visual materials, and the raw CGM data.

The question set was grouped into 6 content domains reflecting topics commonly discussed in diabetes consultations in which CGM review forms an important component: (1) blood glucose interpretation and fluctuation analysis (6 questions), (2) impact of food and exercise (7 questions), (3) medication and treatment guidance (4 questions), (4) emotional and psychological concerns (5 questions), (5) long-term goals and motivation (5 questions), and (6) technical issues and device use (4 questions). These domains were selected to reflect the breadth of patient-facing questions that may arise in such consultations, ranging from direct interpretation of glucose patterns to treatment-related, psychosocial, goal-oriented, and device-related concerns [29]. The draft question bank was reviewed by senior diabetologist EK, who refined its content and added questions based on issues commonly encountered in routine diabetes consultations. The domains were not intended to depend equally on CGM data: some required direct interpretation of CGM metrics and trends, whereas others drew more on the broader clinical context in which CGM findings are discussed. The full question bank is provided in Multimedia Appendix 3.

For each assigned case, clinicians were asked to answer 12 questions drawn from this bank. To ensure coverage of clinically salient topics, each case included 3 questions from the blood glucose interpretation and fluctuation analysis domain, 3 questions related to the impact of food and exercise, 2 questions on medication and treatment guidance, 2 questions on emotional and psychological concerns, and one question each on long-term goals and motivation and on technical issues and device use. Questions specific to type 1 diabetes or intensive insulin therapy (for example, detailed insulin adjustment questions) were not allocated to type 2 diabetes cases. Across the 12 cases, individual question templates were reused with different vignettes and assigned to different clinicians so that each item was answered in multiple clinical contexts while minimizing overlap in the exact question sets seen by any single clinician. Assignment details and frequency statistics for each question are provided in Multimedia Appendix 4.

In the first phase, all case materials were distributed to clinicians on October 22, 2025, and all clinician-authored responses were received by November 18, 2025. Clinicians were instructed to answer their allocated questions as they would when writing to a patient in routine practice, using plain English and making reasonable assumptions based only on the CGM information and vignette provided. No formal time limit was imposed, but clinicians were asked to respond in a manner consistent with their usual clinical communication style. For each case and question, clinicians entered a single free-text response.

To promote broadly comparable levels of detail while preserving individual clinical expression, clinicians were provided with a nonmandatory guideline suggesting responses of approximately 150‐250 words, with flexibility according to content complexity. No strict word limit was imposed. Final CA responses were generated independently on November 14, 2025, using the locked case materials, question bank, prompt templates, RAG corpus, and prespecified generation configuration. Although some clinician-authored responses had been received by that date, they were not used in any part of CA response generation, including model training, fine-tuning, prompt construction, retrieval corpus development, output comparison, or output revision. The CA did not have access to clinician-authored responses during generation. The use of GPT-5.1 reflected the intention to evaluate the most advanced model available at the time of final CA response generation without changing the underlying case materials, prompts, retrieval corpus, or evaluation procedure. The CA generated one free-text response per case-question pair under the same configuration. No explicit word-count constraint was imposed on CA outputs; response length was determined by the model’s generation process within the API token limit. All CA outputs and clinician responses were exported and stored as separate, anonymized text units, labeled only by case ID and question ID.

The second phase was assigned to clinicians on November 19, 2025, after both clinician-authored and CA-generated response sets had been prepared, and all ratings were received by February 15, 2026. In this phase, the same 6 clinicians rated anonymized responses without being informed of the response source. For each case-question pair, raters were provided with the corresponding original case materials and an anonymized response set that included the CA answer and clinician-authored answers written by other clinicians. Raters did not evaluate their own responses. Source recognizability was assessed empirically through the perceived-source label.

Several design features were used to reduce direct self-rating and single-rater calibration effects. First, each response was rated by 3 clinicians rather than by a single assessor, so quality estimates reflected multirater judgment rather than one individual clinician’s scoring pattern. Second, clinicians were recruited from multiple UK NHS Trusts and were not informed which other clinicians had contributed comparator responses, reducing the likelihood of coordination or recognition of colleague-authored responses. Third, responses were anonymized and labeled only by case and question identifiers during rating. Nevertheless, because the same specialist panel contributed comparator responses in Phase 1 and later rated anonymized responses in Phase 2, the rating panel should not be regarded as fully independent of the comparator-generation process.

During the review process, clarification was also provided to all raters regarding the interpretation of certain evaluation criteria, particularly guideline adherence and actionability, when applied to more explanatory questions. Specifically, raters were advised that guideline adherence should be judged in terms of consistency with appropriate clinical practice even when no guideline was explicitly cited and that actionability should not penalize otherwise strong explanatory responses when the question did not naturally call for specific next-step advice.

Quality was rated on 6 5-point Likert-scale dimensions (1=very poor-5=excellent), including clinical accuracy, guideline adherence, actionability, personalization, communication clarity, and empathy/emotional support. The operational definitions of each quality dimension and the rating scale are summarized in Table 1. Safety was captured with a 3-level flag (0=no safety concerns, 1=minor concern requiring revision before clinical use, and 2=major concern with unsafe or clearly contraindicated advice), and raters were also asked to guess the likely source of each response (“human clinician,” “LLM,” or “not sure”; Table 1). Each response, therefore, received up to 3 sets of quality scores, one safety flag, and one perceived source label for subsequent analysis.

**Table 1.** Quality rating dimensions, safety flag categories, and perceived source labels.

Item	Definition / what raters were asked to consider	Scale/categories
Clinical accuracy	Correctness of CGM^a^ interpretation and use of numerical information (eg, TIR^b^/TBR^c^/TAR^d^, mean glucose, variability indices).	5-point Likert scale (1=very poor-5=excellent)
Guideline adherence	Alignment with established diabetes and CGM practice, including consistency with major CGM and diabetes care guidelines.	5-point Likert scale (1=very poor-5=excellent)
Actionability	Clarity and feasibility of suggested next steps and contingencies for the patient, given the CGM patterns and vignette context.	5-point Likert scale (1=very poor-5=excellent)
Personalization	Explicit use of case-specific details from the vignette and CGM data, rather than generic or template-like advice.	5-point Likert scale (1=very poor-5=excellent)
Communication clarity	Ease of understanding for a layperson, including structure, wording, and avoidance of jargon or ambiguous phrasing.	5-point Likert scale (1=very poor-5=excellent)
Empathy / emotional support	Degree of validation, nonjudgmental tone, acknowledgment of emotional burden, and provision of supportive, encouraging language.	5-point Likert scale (1=very poor-5=excellent)
Safety flag	Presence and severity of safety concerns in the advice provided (eg, unsafe insulin suggestions, failure to respond to very high or low glucose).	0=no safety concerns; 1=minor concern requiring revision before clinical use; 2=major concern with unsafe or clearly contraindicated advice
Perceived source	Rater’s guess about whether the response was written by a human clinician or by the CA^e^.	“Human clinician,” “LLM^f^,” or “Not sure”

a CGM: continuous glucose monitoring.

b TIR: time in range.

c TBR: time below range.

d TAR: time above range.

e CA: conversational agent.

f LLM: large language model.

### Post Hoc Retrieval Audit

Twenty-four case-question pairs were audited, with 4 responses selected from each of the 6 predefined question domains. The audited items were selected using a domain-stratified sequential sampling approach: case-question pairs were reviewed in case order, and items were selected in a rotating manner across domains 1-5 to maximize coverage of the predefined question domains, case IDs, and question IDs. Selection was not based on retrieval score, response quality, or the apparent strength of retrieval-response alignment. The audit was therefore intended to provide structured coverage and transparency across the question taxonomy, rather than a statistically representative estimate of retrieval-response alignment across the full response set.

For each audited item, the retrieval process was reconstructed using the same RAG corpus, recursive character-based chunking strategy, embedding model, FAISS vector index, and top k retrieval setting used in the original system configuration. The audit recorded the case ID, question ID, domain, top-ranked source document, retrieved text segment, archived CA response excerpt, FAISS retrieval score, manual retrieval-response alignment category, evidence-to-response role, and rationale for classification. The full item-level audit table is provided in Multimedia Appendix 5.

The retrieval score was the FAISS L2 distance returned by the similarity search function. Lower values indicate greater embedding space proximity between the query and the retrieved segment [55]. This score was used as a quantitative trace of the retrieval process and as a descriptive indicator of why a segment was returned by vector search. It was not treated as a clinical relevance score or as evidence that the retrieved segment necessarily influenced the final CA response. Because embedding space proximity does not directly establish clinical relevance or response grounding, retrieval-response relevance was assessed separately through structured manual review.

Two authors (ZG and KL) independently reviewed the retrieved segment, original question, and archived CA response excerpt for each audited item using a predefined rubric. Retrieved segments were classified as directly aligned, broadly aligned, weakly aligned, or showing no clear alignment with the CA response. Directly aligned indicated that the retrieved text explicitly supported the main claim, explanation, or recommendation in the CA response. Broadly aligned indicated that the retrieved text was relevant to the same clinical topic but provided general contextual support rather than direct evidence for a specific response element. Weakly aligned indicated only indirect or tangential relevance. No clear alignment indicated that no meaningful relationship between the retrieved segment and the question or CA response was visible from the audited materials.

To assess consistency between reviewers, the 4 alignment categories were coded ordinally from 0 to 3, with no clear alignment coded as 0, weakly aligned as 1, broadly aligned as 2, and directly aligned as 3. Interreviewer agreement was assessed using an intraclass correlation coefficient. The agreement between ZG and KL was good (intraclass correlation coefficient [ICC]=0.875). Disagreements were resolved through adjudication by a third author (AL). The apparent role of retrieved evidence was also categorized as directly reflected in the response, conceptually consistent, background or context only, or no clear contribution. This audit was intended to assess retrieval traceability and response alignment, rather than to establish a causal effect of retrieval on response quality.

### Statistical Analysis

Analyses were based on the clinician ratings described above. For each response, 3 raters provided 6 1‐5 quality scores, a 3-level safety flag, and a perceived source label. An overall quality score was defined as the arithmetic mean of the 6 quality items for a given rating. Quality scores for CA and clinician responses were summarized using means, SDs, medians, and IQRs, overall and by question domain. Heatmaps were generated to visualize mean quality ratings across specific domains and dimensions.

Interrater reliability for the quality ratings was evaluated using 2-way random-effects ICCs for absolute agreement [56]. Both single-rater (ICC(2,1)) and average-rater (ICC(2,3)) reliabilities were calculated separately for each quality item and for the overall quality score, with the 3 raters per response treated as interchangeable observers. The 95% CIs were estimated using cluster bootstrap resampling [57] at the response level.

To compare CA and clinician performance, the 1‐5 quality ratings were treated as approximately continuous, consistent with common practice for Likert-type scales in health services research. The primary analysis used linear mixed effects models fitted separately for each quality item and for the overall quality score [58]. In these models, the individual rating was the outcome, responder type (CA vs clinician) was included as a fixed effect with the clinician set as the reference category, and random intercepts for case and rater were included to account for clustering of ratings within CGM cases and systematic differences in individual clinicians’ scoring tendencies. The rater-level random intercept was included to account for potential differences in rating calibration between clinicians, including differences that may have arisen from their clinical background, communication norms, or prior experience of authoring comparator responses in Phase 1. Results were reported as estimated mean differences (CA minus clinician) with 95% CIs and 2-sided *P* values [59]. As a sensitivity analysis, the 3 rater scores for each response were first averaged to create matched case-question pairs. The normality of the paired differences was assessed using the Shapiro-Wilk test [60]. Because the differences deviated from a normal distribution, comparisons between CA and clinician mean scores were conducted using the nonparametric Wilcoxon signed-rank test [61].

To examine whether response length was associated with quality ratings, word count was included as a continuous fixed-effect covariate in additional linear mixed effects models [58]. These models included random intercepts for unique response ID and rater to account for repeated ratings of the same response and systematic differences in rater calibration. An interaction term between responder type and word count was incorporated to assess whether the association between length and quality differed between CA and clinician responses. Statistical significance of the main and interaction effects was evaluated using Wald tests [62].

Domain-specific analyses were conducted to explore whether relative performance varied by clinical topic. For each of the 6 predefined domains, the mixed effects models were refitted on the subset of responses belonging to that domain. Because these analyses involved multiple comparisons and the study was not primarily powered for domain-level hypotheses, domain-specific results are interpreted as exploratory, with an emphasis on effect sizes and CIs rather than formal adjustment for multiplicity.

Safety flags were tabulated for CA and clinician responses and are reported descriptively, given the very low number of flagged responses. For the source-identification task (Turing test [63]), the proportions of responses that raters correctly identified were calculated both overall and at the individual rater level. Identification accuracy was compared against random chance (50%) using exact binomial tests [64]. Furthermore, to assess potential evaluation bias, overall quality scores were stratified and visualized using boxplots based on the raters’ perceived source (ie, whether the rater believed the response was generated by a CA or a clinician), regardless of the true source.

Missing ratings were rare and handled using complete-case analysis without imputation. All statistical tests were 2-sided (except for the one-sided binomial tests assessing accuracy greater than chance), with a significance threshold of *P*<.05. Analyses were conducted using Python 3.11.

### Reporting Framework

This study was a simulated, vignette-based early-stage evaluation of an AI-enabled decision-support and communication-support system, rather than a prospective interventional trial protocol or a randomized clinical trial. To support transparent and reproducible reporting of the AI system and its evaluation, we used the Developmental and Exploratory Clinical Investigations of Decision Support Systems Driven by AI (DECIDE-AI) reporting guideline [65]. DECIDE-AI was developed to guide early-stage clinical evaluations of AI-based decision support systems and was therefore aligned with this study design. The checklist was used to guide reporting of the system’s intended use, intended users, deployment context, input data, model outputs, human oversight requirements, safety boundaries, and potential failure modes. The completed DECIDE-AI checklist is provided in Checklist 1.

## Results

### Rater Characteristics and Interrater Reliability

Six senior diabetes clinicians (male 4 and female 2), each with more than 10 years of postqualification clinical experience, participated in the rating process. A total of 12 CGM-informed diabetes cases were evaluated by the 6 clinicians. Each case comprised 12 structured questions. For every case-question unit, both a CA-generated response and a clinician-authored response were assessed.

In total, 288 unique case-question responses (144 CA and 144 clinician responses) were independently evaluated. Each response was rated by exactly 3 clinicians in a source-masked manner, yielding 864 response-level ratings. Each rating included 6 predefined quality dimensions (clinical accuracy, guideline adherence, actionability, personalization, communication clarity, and empathy), which were averaged to derive an overall quality score for analysis. The rating design was partially crossed but fully balanced at the response level, with no missing data. Details of clinician case assignments are provided in the Methods and Multimedia Appendix 4.

Interrater reliability (Table 2) was assessed using 2-way random-effects ICCs for absolute agreement. For the overall quality score, single-rater reliability was fair (ICC(1,2)=0.272; 95% CI 0.193‐0.342) and increased to a moderate level when averaging 3 raters (ICC(2,3)=0.529; 95% CI 0.418‐0.609). These findings supported the use of aggregated multirater scores for the primary response-level summaries, while indicating variability in individual clinician ratings. Exploratory subgroup analyses examined interrater reliability separately for CA-authored and clinician-authored responses. For overall quality, agreement was numerically higher for CA-authored responses than for clinician-authored responses at both the single-rater level (ICC(1,2) 0.154 vs 0.045; ICC difference 0.109, 95% CI –0.066 to 0.159; *P*=.09) and the averaged-rater level (ICC(2,3) 0.354 vs 0.125; ICC difference 0.229, 95% CI –0.137 to 0.258; *P*=.69), although neither difference was statistically supported.

Across individual quality dimensions, single-rater reliability was generally low to fair, ranging from 0.087 (clinical accuracy; 95% CI 0.007‐0.159) to 0.324 (actionability and empathy; 95% CI 0.245‐0.400 and 0.243‐0.405, respectively). Reliability improved consistently when ratings were averaged across 3 clinicians, with ICC(2,3) values ranging from 0.223 (clinical accuracy; 95% CI 0.022‐0.361) to 0.590 (actionability and empathy; 95% CI 0.493‐0.666 and 0.491‐0.671, respectively).

**Table 2.** Interrater reliability of clinician quality ratings.

Quality dimension	ICC^a^(2,1) (95% CI)		ICC(2,3) (95% CI)	
Clinical accuracy	0.087 (0.007‐0.159)		0.223 (0.022‐0.361)	
Guideline adherence	0.102 (0.023‐0.181)		0.254 (0.065‐0.399)	
Actionability	0.324 (0.245‐0.400)		0.590 (0.493‐0.666)	
Personalization	0.219 (0.141‐0.290)		0.457 (0.330‐0.551)	
Clarity	0.214 (0.140‐0.292)		0.449 (0.328‐0.553)	
Empathy	0.324 (0.243‐0.405)		0.590 (0.491‐0.671)	
Overall quality score	0.272 (0.193‐0.342)		0.529 (0.418‐0.609)	

a ICC: intraclass correlation coefficient.

### Quality Comparisons

Overall quality ratings were compared between CA-generated and clinician-generated responses. Descriptive statistics were calculated using response-level scores averaged across 3 clinician raters, whereas mean differences and CIs were estimated from linear mixed effects models. Across 288 unique case-question responses, CA responses received higher overall quality scores than clinician responses (Table 3). The mean overall quality score was 4.37 (SD 0.57) for CA responses and 3.58 (SD 0.90) for clinician responses. The estimated mean difference was 0.782 (95% CI 0.692‐0.872; *P*<.001). Median scores were also higher for CA responses (4.5, IQR 4.0‐4.8) than for clinician responses (3.8, IQR 3.0‐4.2), indicating a consistent shift in central tendency. Variability differed between response types, with lower dispersion observed for CA scores (SD 0.57) relative to clinician scores (SD 0.90); this pattern suggests lower between-response dispersion in CA ratings.

**Table 3.** Overall and dimension-specific quality ratings for conversational agent (CA) and clinician responses. CA and clinician responses were rated on 6 quality dimensions (1-5), with the overall quality score defined as the mean of the 6 items.

Outcome	CA^a^ mean (SD)	CA median (IQR)	Clinician mean (SD)	Clinician median (IQR)	Mean difference (95% CI)^b^	*P* value
Overall quality	4.37 (0.57)	4.5 (4.0‐4.8)	3.58 (0.90)	3.8 (3.0‐4.2)	0.782 (0.692‐0.872)	<.001
Clinical accuracy	4.40 (0.69)	4.0 (4.0‐5.0)	3.84 (0.95)	4.0 (3.0‐4.0)	0.562 (0.463‐0.662)	<.001
Guideline adherence	4.31 (0.73)	4.0 (4.0‐5.0)	3.82 (0.92)	4.0 (3.0‐4.0)	0.495 (0.394‐0.597)	<.001
Actionability	4.42 (0.71)	5.0 (4.0‐5.0)	3.43 (1.13)	4.0 (3.0‐4.0)	0.992 (0.877‐1.106)	<.001
Personalization	4.25 (0.75)	4.0 (4.0‐5.0)	3.38 (1.11)	4.0 (3.0‐4.0)	0.867 (0.749‐0.985)	<.001
Clarity	4.44 (0.65)	4.0 (4.0‐5.0)	3.73 (1.04)	4.0 (3.0‐5.0)	0.713 (0.611‐0.815)	<.001
Empathy	4.37 (0.65)	4.0 (4.0‐5.0)	3.30 (1.19)	3.0 (3.0‐4.0)	1.062 (0.948‐1.177)	<.001

a CA: conversational agent.

b Mean differences (CA − clinician) were estimated using linear mixed effects models with random intercepts for case and rater.

**Figure 2.**
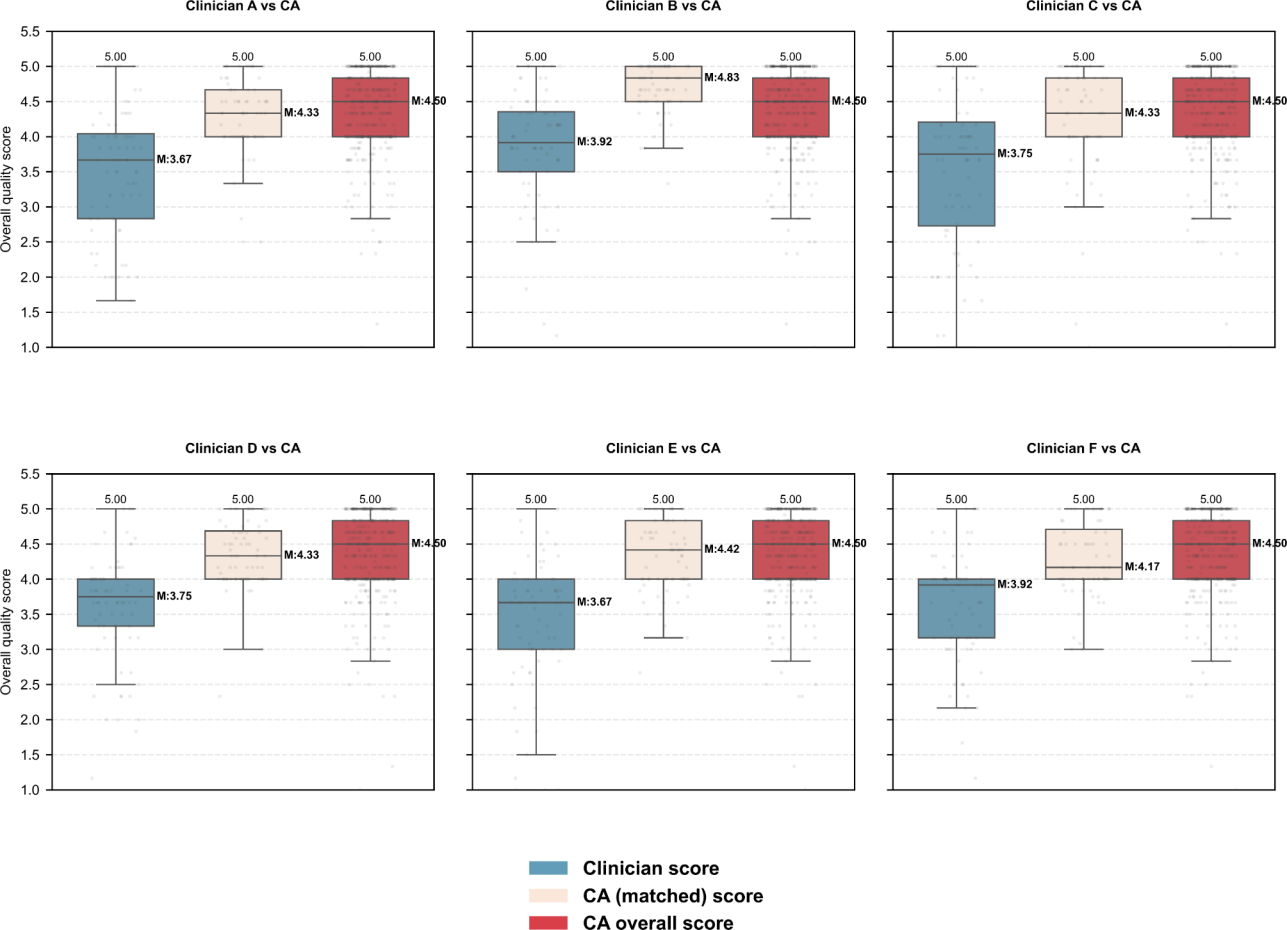
Overall quality score distributions for clinician-authored and conversational agent (CA) responses. Each panel corresponds to one clinician (A-F) and compares clinician-authored responses, CA responses matched to the same cases and questions, and CA responses across all evaluated case-question pairs. Overall quality scores were calculated as the mean of 6 evaluation dimensions. “M” denotes the median; boxes represent the IQR, whiskers indicate the observed range, and points represent individual response-level scores. CA: conversational agent.

To examine whether this overall pattern was consistent across clinician-authored responses, Figure 2 presents overall quality score distributions stratified by the clinician who authored the human responses. In each stratum, the distribution of CA scores was shifted upward relative to clinician-authored responses. CA scores restricted to the same case subsets (“matched”) were comparable to the overall CA distribution, indicating that the observed difference was not confined to particular case allocations.

In sensitivity analyses using response-level matched case–question pairs (n=144), the distribution of paired differences deviated from normality (Shapiro-Wilk *P*=.001). The Wilcoxon signed-rank test confirmed significantly higher scores for CA responses (W=479; *P*<.001), with a median difference of 0.82 points. The estimated rank-biserial correlation (r≈0.95) indicated a large effect size.

Response length differed markedly between CA and clinician responses. The mean word count was 211.4 (SD 54.8; 95% CI 202.4‐220.4) for CA responses compared with 72.9 (SD 68.8; 95% CI 61.6‐84.2) for clinician responses, representing nearly a 3-fold difference in verbosity. Substantial variability in response length was also observed across individual clinicians. Mean word count ranged from 39.5 (SD 15.4; 95% CI 33.0‐46.0) to 191.3 (SD 89.2; 95% CI 153.6‐228.9). Interclinician variability was statistically significant (Kruskal-Wallis H=65.83 [66]; *P*<.001; ε²=0.44) and remained significant after excluding the highest-verbosity clinician (H=20.70; *P*<.001).

Despite these marked differences in response length, word count was not significantly associated with overall quality ratings in mixed effects models (Multimedia Appendix 6). Neither the main effect of word count nor its interaction with responder type was statistically significant for the overall score (all *P*>.14). Among individual quality dimensions, only empathy demonstrated a significant interaction between word count and responder type (*P*=.003). In clinician-authored responses, word count was negatively associated with empathy ratings (β=−0.00235 per word; *P*<.001), whereas no significant association was observed for CA responses (β=0.00107; *P*=.22). Overall, we did not find evidence that word count explained the observed differences in quality between CA and clinician responses.

Dimension-specific mixed effects analyses identified statistically significant differences between CA and clinician responses across all 6 quality dimensions (all *P*<.001). Estimated mean differences ranged from 0.495 to 1.062 (Table 3). The largest differences were observed for empathy (1.062; 95% CI 0.948‐1.177) and actionability (0.992; 95% CI 0.877‐1.106), whereas smaller differences were observed for clinical accuracy (0.562; 95% CI 0.463‐0.662) and guideline adherence (0.495; 95% CI 0.394‐0.597). All estimated mean differences were positive.

Across the 6 predefined content domains, mixed effects models likewise indicated statistically significant differences in overall quality between CA and clinician responses (all *P*≤.025; Multimedia Appendix 7). Estimated mean differences ranged from 0.419 to 1.013. The largest estimated difference was observed in Domain A (blood glucose interpretation and fluctuation analysis; mean difference 1.013, 95% CI 0.762‐1.265), followed by Domain C (medication and treatment guidance; 0.912, 95% CI 0.657‐1.168).

Notably, significant differences were also observed in domain 4, which comprised emotional and psychological concerns (eg, stress-related glycemic dysregulation, fear of hypoglycemia, and feelings of frustration or self-doubt; estimated mean difference 0.721, 95% CI 0.507‐0.935; *P*<.001). In this psychosocial domain, CA responses were consistently rated higher across quality dimensions, including empathy and actionability, indicating that performance differences were not confined to technical CGM interpretation but extended to emotionally sensitive scenarios.

**Figure 3.**
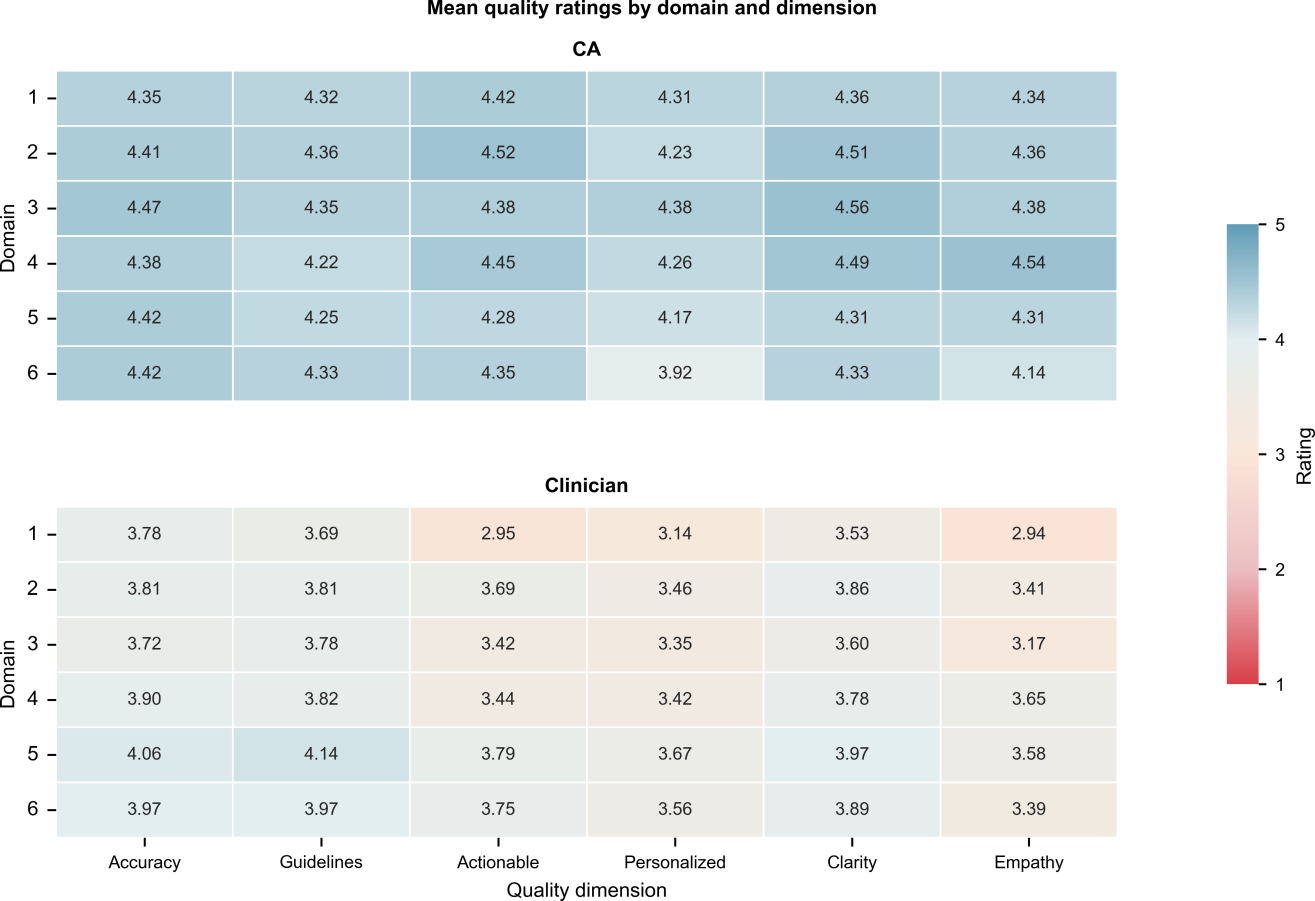
Mean quality ratings by clinical domain and dimension. Heatmaps compare conversational agent (CA)–generated and clinician-generated responses across 6 clinical domains and 6 quality dimensions. Values represent domain-level means of response-level scores averaged across 3 clinician ratings, and color intensity indicates the mean rating on a 1-5 scale. CA: conversational agent.

The smallest estimated differences were observed in domain 5 (long-term goals and motivation; 0.419, 95% CI 0.051‐0.786) and domain 6 (technical issues and device use; 0.493, 95% CI 0.065‐0.921). Figure 3 visualizes the domain- and dimension-specific mean quality ratings, illustrating a consistent pattern of higher scores for CA responses across all 6 content domains and quality dimensions. Full descriptive statistics (mean, SD) are provided in Multimedia Appendix 8.

### Post Hoc Retrieval Audit

A post hoc retrieval audit (Multimedia Appendix 5) was conducted on 24 domain-stratified CA responses, including 4 case-question pairs from each of the 6 predefined question domains. As described in the Methods, the audit sample was selected to maximize domain and case-question coverage rather than to provide a statistically representative estimate of retrieval-response alignment across all CA responses. Across the audited examples, retrieved segments were directly aligned with the corresponding CA response in 8 out of 24 examples (33.3%), broadly aligned in 7 out of 24 (29.2%), weakly aligned in 5 out of 24 (20.8%), and showed no clear alignment in 4 out of 24 (16.7%). Thus, 15 out of 24 audited responses (62.5%) showed direct or broad retrieval-response alignment, while 20 out of 24 (83.3%) showed at least weak topical or contextual relevance. Conversely, 9 out of 24 (37.5%) audited examples showed only weak or no clear alignment, indicating that visible retrieval-response alignment was incomplete and varied across domains.

The mean FAISS L2 distance across audited items was 0.988. Lower L2 distances were directionally associated with stronger manual alignment categories, although the retrieval score was not treated as a standalone relevance measure. The mean L2 distance was 0.855 for directly aligned examples, 0.848 for broadly aligned examples, 1.159 for weakly aligned examples, and 1.288 for examples with no clear alignment. This pattern suggests that embedding-space proximity provided a useful quantitative retrieval trace, but manual review remained necessary to determine clinical relevance and visible response grounding.

Domain-level patterns showed the strongest retrieval-response alignment in blood glucose interpretation and fluctuation analysis, where all 4 audited examples were directly aligned. Medication and treatment guidance and emotional and psychological concerns also showed relatively strong alignment, with direct or broad alignment in 3 of 4 examples in each domain. Long-term goals and motivation showed at least weak alignment in all 4 examples, although only 2 of 4 were directly or broadly aligned. Alignment was weakest for technical issues and device use, where only 1 of 4 examples showed direct or broad alignment and 2 of 4 showed no clear alignment.

### Safety and Source Identification

Beyond quantitative quality comparisons, we examined raters’ ability to identify the source of each response and the distribution of safety flags.

Across 864 ratings, clinicians correctly identified the source in 697 (80.7%) cases, misclassified 100 (11.6%), and selected “not sure” in 67 (7.8%). Restricting the analysis to definitive judgments (n=797 rating-level classifications), overall identification accuracy was 87.5% (one-sided exact binomial test vs 50% chance, *P*<.001), indicating that responses were generally distinguishable from one another. These findings indicate that, although the response source was masked during the rating task, CA and clinician responses were often distinguishable in practice.

Identification performance varied across raters. 5 clinicians demonstrated high discrimination accuracy (82.3%‐100.0% among definitive judgments), whereas one clinician did not perform above chance level (50.0%; *P*=.54), suggesting substantial interrater heterogeneity. Rater-specific distributions of overall quality scores, stratified by perceived source (clinician vs CA), are shown in Multimedia Appendix 9. Descriptively, responses perceived as CA tended to receive higher median quality scores for most raters, although this pattern was not uniform and some raters showed similar or lower scores for responses perceived as CA. This analysis was exploratory and was not interpreted as a causal test of source-label bias because perceived source, true source, response style, and response structure were closely entangled.

Safety flag distributions were comparable between sources. Among clinician responses (n=432), 387 (89.6%) were rated as Level 0, 42 (9.7%) as Level 1, and 3 (0.7%) as Level 2. Among CA responses (n=432), corresponding proportions were 389 (90.1%), 40 (9.3%), and 3 (0.7%), respectively. However, qualitative comments were available for only 23 flagged ratings, as written explanations were optional, and therefore not all safety flags were accompanied by narrative justification.

Among the 23 available comments, most (n=15, 65.2%) concerned glucagon-like peptide-1 (GLP-1) eligibility under NHS BMI criteria, with most of these applying to CA responses (13 CA vs 2 clinicians). Three (13.0%) comments related to medication administration, specifically acarbose dosing (2 CA vs 1 clinician), and 2 (8.7%) comments noted that the response did not directly address the question. The remaining comments (n=3, all CA) referred to behavioral feasibility concerns, including the perceived burden of the proposed action plan and the appropriateness of specific dietary substitution advice. These comment-level data should be interpreted cautiously because written remarks were optional, but they suggest that similar flag frequencies do not necessarily imply identical patterns of concern across response sources.

## Discussion

### Principal Findings

In this source-masked multirater evaluation of vignette-based CGM-informed scenarios, the retrieval-grounded CA received higher mean structured quality ratings than clinician-authored responses across predefined domains. The overall mean difference was approximately 0.8 points on a 5-point scale, with the largest differences observed in empathy (mean difference 1.06) and actionability (0.99). These findings suggest that, under standardized vignette-based conditions, a scaffolded CA output pipeline incorporating retrieval as part of its architecture can produce written responses that specialists judge favorably in both relational and action-oriented dimensions. However, the comparison should be interpreted as a controlled evaluation of written outputs rather than as evidence that the CA is intrinsically superior to clinicians in real-world diabetes counseling. To our knowledge, this represents one of the first source-masked comparative evaluations of retrieval-grounded LLM-generated and clinician-authored responses in structured CGM counseling contexts.

Performance differences were not uniform across domains and appeared to vary according to task structure. Differences were most pronounced in data-intensive glucose interpretation and action-oriented explanation and attenuated in domains centered on long-term motivational support and device troubleshooting. Notably, significant differences were also observed in psychosocial scenarios, indicating that comparative ratings were not limited to quantitative glucose analysis but extended to contexts involving emotional distress, fear of hypoglycemia, and diabetes-related frustration. This gradient suggests that relative performance may depend on the cognitive structure of the task. The scaffolded CA pipeline evaluated here appeared particularly well suited to synthesizing numerical CGM metrics, vignette context, structured prompting, and guideline-oriented reference material into structured explanations and action plans [25]. In contrast, domains requiring nuanced behavioral coaching or experiential clinical judgment may depend more heavily on individualized framing [67,68].

The post hoc retrieval audit provides additional context for interpreting the contribution of the RAG component. The audit suggested that retrieval grounding was partial and domain-dependent rather than uniform across all CA outputs. Retrieval-response alignment was strongest for blood glucose interpretation and fluctuation analysis, where all audited examples were directly aligned, and was also relatively strong for medication and treatment guidance and emotional and psychological concerns. This pattern suggests that the curated corpus was most useful when questions could be supported by guideline-based or educational material directly relevant to CGM interpretation, treatment-context explanation, or psychosocial support. In contrast, technical issues and device use showed weaker visible retrieval-response alignment, suggesting that future versions may require more targeted device-specific retrieval materials, such as manufacturer guidance, sensor troubleshooting documentation, and local clinical protocols. Therefore, the RAG component should be interpreted as providing retrieval traceability and visible response alignment in a subset of cases, rather than as uniformly determining CA outputs or explaining the observed quality differences.

### Comparison With Prior Work

These findings extend prior CGM-focused evaluations of LLM-generated summaries, which have typically assessed model outputs in isolation and emphasized feasibility, accuracy, or clinical acceptability [31]. By using a balanced design with source-masked ratings, this study enables direct comparison with clinician-authored responses across predefined technical and relational dimensions. Notably, the largest differences were observed in actionability and empathy, rather than being confined solely to glycemic interpretation, suggesting that structured explanatory and relational components may be particularly sensitive to comparative evaluation.

Related work in other wearable-data domains has explored the use of AI systems to interpret structured physiological data streams, such as heart rate, physical activity, or sleep measures generated by consumer wearables [69-71]. These studies have similarly focused on automated summarization, health insight generation, or behavioral coaching based on wearable-derived data [70]. However, most have evaluated system outputs in isolation or against guideline-based expectations rather than through source-masked comparison with clinician-authored responses. This study therefore contributes additional evidence by examining how LLM-generated explanations compare directly with clinician communication in a structured evaluation setting.

The evaluation was confined to structured, vignette-based scenarios involving common CGM-related questions, concept clarification, and general diabetes self-management guidance. It did not assess complex therapeutic decision-making, individualized prescribing adjustments, or real-time risk management. Accordingly, these findings should be interpreted within the scope of clearly bounded explanatory tasks, rather than extended to higher-stakes clinical reasoning contexts.

Beyond CGM-specific evaluations, these findings align with broader diabetes care literature, suggesting cautious optimism regarding AI-supported communication tools. Reported areas of potential utility include patient education, explanation of structured health data, and information synthesis, whereas greater caution is typically expressed when systems are used to support individualized treatment decisions or medication adjustments [68,72]. Within this context, scaffolded CAs incorporating retrieval may be best viewed as adjunct tools for patient-facing explanation in clearly defined, guideline-constrained use cases, with clinician oversight maintained for interpretation and final clinical framing [31,67-72].

Although workflow outcomes were not directly evaluated, the observed performance in standardized CGM explanation tasks suggests potential relevance to clinical workflow. In routine practice, summarizing CGM trends, clarifying commonly used thresholds, and addressing frequent educational queries can consume substantial consultation time [46]. If deployed within appropriately bounded tasks and aligned to local guidance and policy constraints, scaffolded CAs incorporating retrieval may help support these informational components of care. Any such use should be framed as supportive rather than substitutive, and prospective studies are needed to quantify effects on clinician workload, consultation flow, and patient outcomes.

### Limitations

Several factors limit how these findings should be interpreted and generalized. First, although the study incorporated safeguards against direct self-rating and single-rater effects, the clinician panel was not fully independent of the comparator-generation process because the same group of specialists authored comparator responses in Phase 1 and rated anonymized responses in Phase 2. These safeguards reduce the risk of direct self-rating and idiosyncratic single-rater judgments, but they do not remove the possibility that clinicians brought expectations from their own authoring experience into the rating phase. Interrater agreement was also modest, with single-rater reliability ranging from poor to fair across most dimensions (ICC(1,2) range: 0.087‐0.324). This likely reflects the difficulty of rating complex patient-facing clinical narratives, where expert judgments may vary according to clinical training background, subspecialty experience, local practice norms, communication style, and individual interpretation of rating dimensions such as guideline adherence, actionability, and empathy [73-75]. Reliability improved when ratings were averaged across 3 clinicians, supporting the use of aggregated multirater scores for primary interpretation. However, dimension-specific findings, particularly those based on modest absolute differences, should be interpreted cautiously because the rating instrument and sample size may not support strong inferences at the individual-dimension level.

Second, the case set was small and included only 3 type 2 diabetes cases. This limits generalizability to routine diabetes care, where type 2 diabetes accounts for the majority of diagnosed diabetes [76]. However, this imbalance also reflects a broader data-availability constraint, as CGM use and publicly available CGM datasets remain disproportionately concentrated in type 1 diabetes and insulin-treated populations [77,78]. More broadly, the study had limited coverage of medication-policy-sensitive scenarios, including questions involving treatment escalation, eligibility criteria, cardiometabolic risk management, and locally specific prescribing rules. At the aggregate level, safety flag frequencies were similar between CA and clinician responses, and major safety concerns were rare. However, optional written safety comments were more often attached to CA responses (n=20, 87%) and most frequently concerned GLP-1 eligibility under NHS BMI criteria [74]. This should be interpreted primarily as a local prescribing and policy-alignment issue rather than as a general CGM interpretation error. It also suggests that similar aggregate safety flag frequencies should not be interpreted as identical safety profiles across response sources, diabetes types, or treatment contexts. Larger studies with more diverse type 1 and type 2 diabetes cases, including different treatment regimens, BMI profiles, medication eligibility scenarios, and local prescribing policies, are needed before extending these findings to routine diabetes care.

Third, the comparison should be interpreted in light of imperfect source masking, response recognizability, and potential development-stage familiarity bias. Although raters were not informed of the response source and all responses were anonymized, source-identification accuracy was high, indicating that the evaluation was source-masked by design but imperfectly blinded in practice. This recognizability likely reflected deliberate features of the CA configuration, including structured prompting, safety-bounded generation, plain-language communication guidance, and a low temperature setting intended to reduce stochastic variation and improve reproducibility [79]. These choices were intended to derisk patient-facing communication, but they may also have increased the consistency and recognizability of CA outputs.

In addition, one senior diabetologist provided formative feedback during vignette and question-bank development and later participated as one of the clinician raters. This creates potential circularity or familiarity bias, particularly regarding expectations about appropriate response structure, tone, or clinical framing. This risk was reduced because the pilot profile reviewed during development was not included in the final case library, development-stage response excerpts were not rated as formal outputs, and the final evaluation used a source-masked multirater design involving 6 clinicians. However, these safeguards do not fully remove the possibility that response style, source recognizability, or development-stage familiarity influenced ratings, particularly for empathy and personalization.

The perceived-source analysis suggested rater-specific heterogeneity: responses perceived as CA often received higher scores, but this pattern was not consistent across all raters, indicating that perceptual bias, if present, was unlikely to operate uniformly. Personalization ratings may also have been affected by the CA’s more explicit use of case-specific CGM and vignette details, whereas clinicians may have conveyed individualized judgment more concisely or implicitly. Although word-count-adjusted analyses did not indicate that response length explained the overall quality difference, response style and recognizability may still have influenced how raters applied relational criteria. Higher empathy ratings for CA responses should therefore be interpreted as higher perceived empathy of the written outputs under this evaluation design, rather than as evidence of intrinsic empathic capacity or superiority over clinician communication in real-world consultations. Future studies should separate vignette development, system refinement, and outcome rating across independent clinical panels where feasible.

A related limitation is the asymmetry between the scaffolded CA configuration and the clinician-authored response condition. The clinician contributors were senior diabetes specialists with substantial clinical experience, tacit guideline knowledge, and real-world communication expertise. Their responses, therefore, should not be interpreted as reflecting a lack of clinical knowledge or capability, but as usual, expert-authored written communication under the constraints of a vignette-based task. By contrast, the CA was explicitly configured to produce standardized written explanations using structured prompting, communication and safety instructions, RAG-retrieved reference material, and low-temperature generation. These design features may have improved consistency, completeness, structure, and alignment with the study rating rubric, particularly for clarity, actionability, and empathy. Accordingly, the observed quality differences should be interpreted as differences between a scaffolded retrieval-grounded CA output pipeline and usual clinician-authored written responses under controlled vignette conditions, rather than as evidence that the CA is intrinsically superior to clinicians in clinical communication or real-world diabetes counseling. In real clinical practice, clinicians provide interactive clarification, longitudinal knowledge of the patient, contextual judgment, and responsibility for treatment decisions, which were not fully represented in this single-turn written evaluation. Future studies could examine complementary comparison conditions, such as clinician-authored responses supported by structured templates, shared reference materials, or CA-assisted drafting workflows, to distinguish the effect of response scaffolding from the effect of model generation itself.

Fourth, the findings reflect a single retrieval-grounded system and one model configuration. Generation parameters, including the low temperature setting and API access timing, are reported in the Methods to improve reproducibility [79]; however, outputs may differ under alternative models, API snapshots, retrieval resources, chunking strategies, prompts, or generation settings. The post hoc retrieval audit also showed that RAG contribution was partial and domain-dependent rather than uniformly visible across all outputs. Some responses were directly or broadly aligned with retrieved material, whereas others showed only weak or no clear visible contribution from the top-ranked retrieved segment. Importantly, this audit was designed to assess retrieval traceability and visible response alignment, not to establish a causal effect of retrieval on response quality. Without an ablation comparison, this study cannot determine how much of the observed performance reflects retrieval grounding rather than the base model’s parametric knowledge, structured prompting, CGM summary inputs, vignette context, or safety-oriented response instructions. Accordingly, “retrieval-grounded” should be understood here as a description of the system architecture and input pipeline rather than as evidence that retrieval was the primary driver of the observed quality ratings. The weaker alignment observed for technical issues and device use likely reflects the scope of the current corpus, which included limited sensor-use and device-troubleshooting material. Future work should include full retrieval logging, ablation comparisons, independent relevance assessment, and expanded device-specific and local policy-specific retrieval corpora.

Finally, the study used written, vignette-based scenarios rather than interactive real-world consultations. Although vignettes were derived from real CGM data and reviewed for clinical plausibility, they necessarily simplified real-world encounters and did not encompass rare presentations, highly complex comorbidities, diverse treatment pathways, or dynamic patient-clinician interaction. The findings are therefore most applicable to structured CGM interpretation and patient-facing explanation tasks under controlled conditions. Future studies should evaluate scaffolded retrieval-grounded systems prospectively in clinical workflows, such as preconsultation preparation or supervised patient education, and assess patient understanding, consultation efficiency, clinician workload, safety escalation, and local governance requirements.

### Conclusions

Taken together, these findings support a potential role for scaffolded LLM systems incorporating retrieval as part of their architecture as adjunct tools for structured CGM interpretation and patient-facing explanation of common questions arising in routine diabetes care under controlled, source-masked vignette-based conditions. In practical terms, their most appropriate role appears to be explanatory and educational rather than therapeutic: they may help patients understand glucose patterns, clarify common concerns, and prepare for discussion with their diabetes care team, including in relation to emotionally sensitive issues. However, this should not be interpreted as support for individualized therapeutic decision-making. In particular, such systems are not established here as appropriate for recommending medication initiation, dose adjustment, regimen change, or other personalized treatment decisions, which should remain clinician-led. Nor do these findings establish equivalence to clinician communication in real-world consultations, where ongoing therapeutic relationships, contextual judgment, and dynamic interaction remain central. The findings should also be interpreted in light of the imperfect source masking and the asymmetry between scaffolded CA outputs and unassisted clinician-authored responses. Further prospective evaluation in interactive clinical settings is needed to define the appropriate scope, safeguards, and implementation of such systems in practice.

## Supplementary material

10.2196/98519 Multimedia Appendix 1 Example patient profile.

10.2196/98519 Multimedia Appendix 2 Prompt to build the conversational agent.

10.2196/98519 Multimedia Appendix 3 Full question bank.

10.2196/98519 Multimedia Appendix 4 Clinician case assignments, question-ID coverage, and reviewed cases & Frequency of Each Question ID Across All 12 Cases (N=144).

10.2196/98519 Multimedia Appendix 5 Retrieval audit of top-ranked retrieved segments and their relationship to archived clinical assistant responses.

10.2196/98519 Multimedia Appendix 6 Association between response length and quality ratings.

10.2196/98519 Multimedia Appendix 7 Domain-specific mixed effects model results comparing CA and clinician responses.

10.2196/98519 Multimedia Appendix 8 Domain- and dimension-specific quality ratings (mean ± SD).

10.2196/98519 Multimedia Appendix 9 Rater-level distribution of overall quality scores stratified by perceived source.

10.2196/98519 Checklist 1 DECIDE-AI checklist.

## Abbreviations

ADA: American Diabetes Association
AGP: ambulatory glucose profile
CA: conversational agent
CGM: continuous glucose monitoring
DECIDE-AI: Developmental and Exploratory Clinical Investigations of Decision Support Systems Driven by AI
FAISS: Facebook AI Similarity Search
GLP-1: glucagon-like peptide-1
ICC: intraclass correlation coefficient
IDF: International Diabetes Federation
LLM: large language model
NHS: National Health Service
RAG: retrieval-augmented generation
TAR: time above range
TBR: time below range
TIR: time in range
UCLH: University College London Hospitals
