# Supplementary material for: Retrieval-Augmented Large Language Model Counseling for Continuous Glucose Monitoring in Diabetes: Source-Masked Multirater Comparative Evaluation
Source: J Med Internet Res. 2026 Jul 31;28:e98519. doi: 10.2196/98519 (PMC13430954; doi:10.2196/98519)
Supplement: Multimedia Appendix 1 [file jmir-v28-e98519-s001.docx]

**Multimedia Appendix 1**

Patient Name: Steven (ID: 1002)
Date: Based on CGM Data – May 2021

1. Demographics & Medical History

- Age: 36 years
- Sex: Male
- Height / Weight / BMI: 172 cm / 68 kg / BMI: 23.0
- Occupation: Graphic designer (mostly sedentary, 8+ hours/day screen time)
- Living Situation: Lives with partner; meals are prepared at home on weekdays, dine-out on weekends
- Diabetes Type: Type 1 Diabetes Mellitus (diagnosed at age 30)
- Duration: 6 years since diagnosis
- Family History: Father has hypertension; no known family history of type 1 or type 2 diabetes
- Diabetes Education: Attended structured DSME course in 2020; currently self-managing pump and CGM independently

2. Treatment & Medication

- Insulin Regimen:
  - Delivery: Continuous Subcutaneous Insulin Infusion (CSII) – Novolin R
  - Basal Insulin:
    - Mean basal rate: 0.68 IU/h
    - Median basal rate: 0.7 IU/h
  - Bolus Insulin:
    - Total bolus injections recorded: 28
    - Mean bolus dose: 5.46 IU
    - Administered manually before meals based on experience (no auto calculator)
- Other Antihyperglycemics: None
- Adherence:
  - Wears CGM regularly (>89% wear time in recent monitoring)
  - Consistently inputs bolus doses; manages basal profiles without assistance
  - Does not currently count carbohydrates or use an insulin-to-carb ratio (ICR)

3. CGM Monitoring Summary

Monitoring duration: 11 days
CGM wear time: 89.8%
Total glucose readings: 948

| Metric | Value | Recommended Target* |
| --- | --- | --- |
| Mean glucose | 136.0 mg/dL (7.6 mmol/L) | — |
| GMI (Estimated HbA1c) | 6.56% | — |
| Standard deviation | 59.9 mg/dL (3.3 mmol/L) | — |
| Coefficient of variation | 44.0% | <36% |
| Time in Range (70–180 mg/dL [3.9–10.0 mmol/L]) | 74.8% | >70% |
| Time Below Range (<70 mg/dL [<3.9 mmol/L]) | 7.1% | <4% |
| Time in Very Low (<54 mg/dL [<3.0 mmol/L]) | 0.8% | <1% |
| Time Above Range (>180 mg/dL [>10.0 mmol/L]) | 18.1% | <25% |
| Time in Very High (>250 mg/dL [>13.9 mmol/L]) | 5.9% | <5% |

* Based on international CGM consensus targets


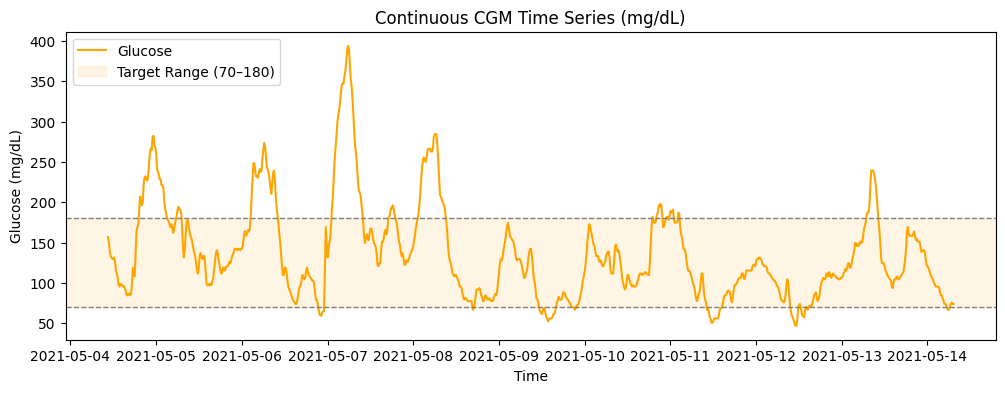


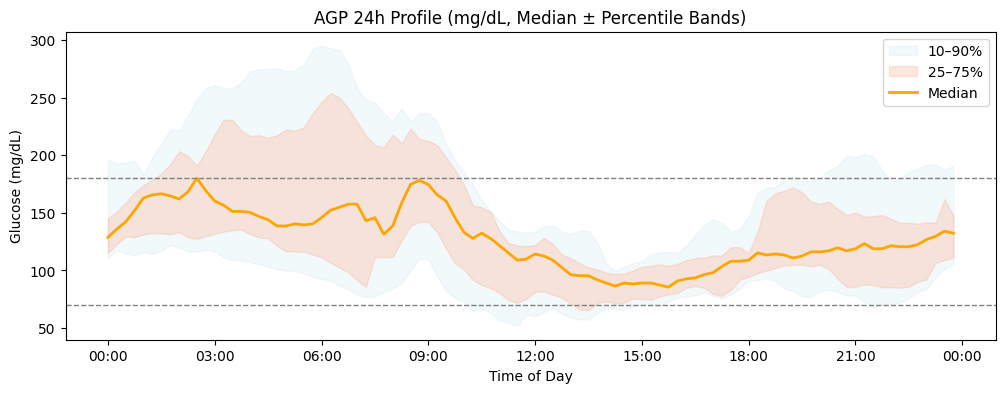


4. Lifestyle & Daily Routine

- Diet:
  - Weekdays: three regular meals at home
    - Breakfast (8:30 AM): oatmeal with milk, egg or toast
    - Lunch (1:00 PM): rice/noodles with vegetables and meat
    - Dinner (7:00 PM): usually stir-fried dishes with rice or soup
  - Weekends: dine out (hotpot, fast food, or noodles), heavier carbohydrate load
  - Occasional afternoon snack (~4 PM): fruit, yogurt, or biscuit
  - Avoids sugary drinks except for hypoglycemia treatment (uses juice or glucose tabs)
  - Alcohol: ~1–2 drinks per month, typically with meals
  - Does not smoke
- Physical Activity:
  - Walks 15–20 minutes daily (commute and errands)
  - No structured exercise routine
  - Does not pre-adjust insulin for exercise; monitors symptoms and eats snacks as needed
- Sleep:
  - Sleep from ~12:30 AM to 7:30 AM on weekdays
  - Slightly longer on weekends
  - Occasionally reports post-lunch fatigue
- Stress & Work:
  - Moderate work stress due to deadlines
  - Seated >8 hours daily due to nature of desk job

5. Self-Management Behavior

- Insulin Administration:
  - Comfortable using CSII independently
  - Adjusts basal settings when necessary (e.g., illness, heavy meals)
  - Does not use bolus calculator; estimates boluses based on food size/type
  - Fingerstick glucose checks: 2–3 times/week, especially to confirm CGM readings <70 mg/dL (<3.9 mmol/L) and to rule out falsely low readings
- Monitoring Practices:
  - Uses FreeStyle Libre daily; scans CGM at least every 8 hours
  - Uploads data to LibreView before appointments
  - Occasionally performs fingerstick blood glucose (2–3 times/week), especially when feeling low
- Record Keeping:
  - No written food logs
  - No use of mobile apps for dose tracking or meal logs
  - No documented use of ICR/ISF ratios or correction bolus formulas
- Education Status:
  - Completed diabetes self-management education once
  - No recent refresher but open to follow-up sessions
  - Has not seen a dietitian in past 2 years

6. Reported Challenges & Self-Observations

- Notices afternoon lows (~3–4 PM), especially on days with walking or delayed lunch
- Experiences occasional spikes after weekend meals
- Sometimes fatigued in late morning or post-lunch
- No severe hypoglycemia requiring assistance
- Finds carb counting tedious; prefers consistent meals to simplify insulin dosing
- Would like to improve post-meal stability, especially after lunch and weekend dinners

7. Clinical Readiness & Patient Engagement

The patient demonstrates high engagement with diabetes technology and self-care routines. He uses a CGM regularly with >89% wear time and inputs insulin doses consistently via CSII. He monitors glucose fluctuations and is comfortable adjusting basal rates manually.

Although he does not currently practice carbohydrate counting or use an ICR/ISF model, he maintains stable daily routines and reports meal-based decision-making. He performs occasional capillary glucose checks in specific situations (e.g., symptoms, driving) and uploads CGM data proactively.

He does not maintain written logs of meals or insulin, but his meal patterns are regular. He is receptive to additional education or nutrition counseling and is interested in improving day-to-day glucose stability without complicating his routine. No behavioral red flags or self-care barriers are currently reported.
