# Supplementary material for: Retrieval-Augmented Large Language Model Counseling for Continuous Glucose Monitoring in Diabetes: Source-Masked Multirater Comparative Evaluation
Source: J Med Internet Res. 2026 Jul 31;28:e98519. doi: 10.2196/98519 (PMC13430954; doi:10.2196/98519)
Supplement: Multimedia Appendix 3 [file jmir-v28-e98519-s003.docx]

**Multimedia Appendix 3**

**A: Blood Glucose Interpretation & Fluctuation Analysis**

1. “Are my glucose readings normal? Why do they fluctuate like this?”
2. “Why does my blood sugar spike after breakfast?”
3. “Is my TIR good enough?”
4. “Why do I drop low during the night even when I sleep normally?”
5. “My blood sugar is all over the place this week, what’s going on?”
6. ‘‘Whatever I do, I cannot bring down my fasting blood sugar.’’

**B: Impact of Food & Exercise**

1. “I ate or exercised this way， but why did my blood sugar change like that?”
2. “I had noodles and my sugar shot up to 13, should I avoid carbs completely?”
3. “Should I exercise before or after meals?”
4. “Are low-calorie or zero-sugar drinks actually safe?”
5. ‘‘I cannot remember to bolus before meals, I do not always know if I am going to eat within the next 20 minutes.’’
6. ‘‘I often have hypos after meals, why does this happen?’’
7. ‘‘ What to do with alcohol?’’

**C: Medication & Treatment Guidance**

1. “Is my medication working? Do I need to change anything?
2. “I’m taking meds, but my sugar is still high, why?”
3. “Am I going to need insulin soon?”
4. “Could it be that I’m taking my pills too early or late?”

**D:** **Emotional & Psychological Concerns**

1. “My stress or mood affects my sugar, what should I do?”
2. “I’ve been under a lot of stress and my blood sugar is a mess.”
3. “I’ve worked so hard but my glucose is still bad, I feel defeated.”
4. “Other people are doing better than me. Am I failing?”
5. ‘‘I am very afraid of hypos and I do less insulin than I should.’’

**E: Long-term Goals & Motivation**

1. “Can I really get better? What should I aim for?”
2. “I want my HbA1c below 6.5%, how can I achieve it?”
3. “Is there any chance I can get off medications?”
4. “Once I start insulin, is it forever?”
5. ‘‘I definitely want to avoid complications, but I find it hard to achieve 100% TIR.’’

**F:** **Technical Issues & Device Use**

1. “Is my CGM device working properly?”
2. “Is this spike real, or is the sensor wrong?”
3. “My sensor keeps falling off, what can I do?”
4. “Why is there a gap in my readings last night?”
