# Supplementary material for: Retrieval-Augmented Large Language Model Counseling for Continuous Glucose Monitoring in Diabetes: Source-Masked Multirater Comparative Evaluation
Source: J Med Internet Res. 2026 Jul 31;28:e98519. doi: 10.2196/98519 (PMC13430954; doi:10.2196/98519)
Supplement: Multimedia Appendix 4 [file jmir-v28-e98519-s004.docx]

**Multimedia Appendix 4**

**S 1: Clinician case assignments, question-ID coverage, and reviewed cases**

| **Clinician** | **Completed Cases** | **Question IDs Covered in Completed Cases** | **Cases Reviewed (6 per clinician)** |
| --- | --- | --- | --- |
| **D1** | Case 1, Case 7 | **Case 1:** A1, A2, A3, B1, B3, B5, C1, C2*, D1, D5, E1, F1 **Case 7:** A4, A5, A6, B2, B4, B6*, C3, C4, D2, D3, E2, F3 | Case 2, Case 3, Case 4, Case 5, Case 6, Case 8 |
| **D2** | Case 2, Case 8 | **Case 2:** A1, A5, A6, B5, B6, B7, C1, C4, D1, D4, E3, F4 **Case 8:** A2, A4, A5, B2, B3, B4, C2, C3, D2, D3, E2, F2 | Case 1, Case 3, Case 4, Case 7, Case 10, Case 12 |
| **D3** | Case 3, Case 9 | **Case 3:** A3, A4, A5, B3, B4, B5, C1, C4, D3, D5, E4, F3 **Case 9:** A1, A2, A6, B1, B6, B7, C3, C2, D2, D4, E3, F1 | Case 1, Case 2, Case 6, Case 7, Case 8, Case 11 |
| **D4** | Case 4, Case 10 | **Case 4:** A4, A5, A6, B1, B2, B7, C1, C4, D4, D5, E4, F4 **Case 10:** A1, A2, A3, B1, B6, B7, C2, C3, D1, D2, E5, F2 | Case 1, Case 5, Case 6, Case 9, Case 11, Case 12 |
| **D5** | Case 5, Case 11 | **Case 5:** A1, A5, A6, B1, B2, B3, C1, C4, D1, D3, E5, F1 **Case 11:** A3, A4, A5, B2, B4, B6, C2, C3, D2, D4, E1, F3 | Case 2, Case 3, Case 7, Case 9 Case 10, Case 12 |
| **D6** | Case 6, Case 12 | **Case 6:** A1, A2, A6, B1, B4, B5, C2, C4, D1, D3, E1, F2 **Case 12:** A3, A4, A5, B3, B6, B7, C1, C3, D3, D4, E2, F4 | Case 4, Case 5, Case 8, Case 9, Case 10, Case 11 |

**S 2: Frequency of each question ID across all 12 cases (N = 144)**

| **Question ID** | **Count** | **Question ID** | **Count** |
| --- | --- | --- | --- |
| **A1** | 6 | **C1** | 6 |
| **A2** | 5 | **C2** | 6 |
| **A3** | 5 | **C3** | 6 |
| **A4** | 6 | **C4** | 6 |
| **A5** | 8 | **D1** | 5 |
| **A6** | 6 | **D2** | 5 |
| **B1** | 6 | **D3** | **6** |
| **B2** | 5 | **D4** | 5 |
| **B3** | 5 | **D5** | 3 |
| **B4** | 5 | **E1** | 3 |
| **B5** | 4 | **E2** | 3 |
| **B6** | 6 | **E3** | 2 |
| **B7** | 5 | **E4** | 2 |
|  |  | **E5** | 2 |
| **F1** | 3 | **F3** | 3 |
| **F2** | 3 | **F4** | 3 |
