# Supplementary material for: Retrieval-Augmented Large Language Model Counseling for Continuous Glucose Monitoring in Diabetes: Source-Masked Multirater Comparative Evaluation
Source: J Med Internet Res. 2026 Jul 31;28:e98519. doi: 10.2196/98519 (PMC13430954; doi:10.2196/98519)
Supplement: Multimedia Appendix 5 [file jmir-v28-e98519-s005.docx]

**Multimedia Appendix 5**

**Retrieval audit of top-ranked retrieved segments and their relationship to archived clinical assistant responses.**

| **Case ID** | **Question ID** | **Domain** | **Top-ranked source** | **Page** | **Rank 1 retrieved guideline segment** | **Archived CA response** | **Retrieval score (L2 distance)** | **Relationship** | **Role** | **Rationale for classification** |
| --- | --- | --- | --- | --- | --- | --- | --- | --- | --- | --- |
| 1 | A1 | A | agp_interpretation.pdf | 5 | ..glucose variability is caused by occasional factors, such as unplanned meals and snacks, or intermittent exercise... | ...Your data shows notable glucose fluctuations. As outlined in clinical interpretation guidelines, such variability is frequently tied to daily factors like unplanned meals or exercise routines. To better stabilize your levels | 0.733 | Directly aligned | Directly reflected in response | The retrieved segment links glucose variability to meals, snacks, and exercise, which is directly repeated in the CA explanation. |
| 1 | B1 | B | Standards of Care in.pdf | 10 | ...Intense activities may actually raise blood glucose levels instead of lowering them... variation in glycemic response to exercise bouts… | ...You currently handle exercise by reacting to symptoms with snacks rather than managing insulin proactively. Without preventative measures, your glycemic response can vary, causing blood glucose to fluctuate in unpredictable ways during activity… | 0.433 | Directly aligned | Directly reflected in response | The retrieved segment discusses exercise-related glucose variability, and the CA response uses the same mechanism. |
| 2 | C1 | C | Diabetes UK Australian Handbook.pdf | 184 | ...your medication(s)? Have you noticed any changes in how you feel – positive or negative?’ Some psychotropic medications... | ...Your current setup using Regular insulin in the pump starts slowly and lingers longer, impacting your post-meal control and causing late dips... | 0.693 | Broadly aligned | Conceptually consistent | The retrieved segment concerns medication-related glycemic effects, matching the treatment focus, but not the specific insulin pharmacodynamics. |
| 2 | D1 | D | Diabetes UK Australian Handbook.pdf | 67 | ...suggestions that the person may try, in order to reduce their distress, and offers suggestions for support and additional information.. | ...When you’re stressed or sleep deprived, your body releases adrenaline and cortisol, which push sugar from… | 0.897 | Broadly aligned | Background/context only | The retrieved segment supports the psychosocial context, but not the specific cortisol/adrenaline mechanism. |
| 3 | E4 | E | Diabetes UK Australian Handbook.pdf | 197 | ...continue activities after starting insulin. • Explain how to titrate insulin doses based on blood glucose readings and planned activities and meals, to avoid .... | ...With type 1 diabetes, insulin isn’t a punishment or a sign of failure—it’s simply the hormone your body can’t make enough of anymore.... | 0.778 | Broadly aligned | Conceptually consistent | The retrieved segment concerns insulin use and glucose management, while the CA response applies this topic in a motivational context. |
| 3 | F3 | F | agp_interpretation.pdf | 1 | ...during which it automatically takes and stores a glucose reading every minute. Glucose values are transmitted either to a dedicated reader... | ...it drops suddenly to a flat line and pops back up when you shift positions, it occurs during the first 24 hours of a new sensor.... | 0.812 | Broadly aligned | Background/context only | The retrieved segment is device-relevant, but does not directly support the specific sensor artefact explanation. |
| 4 | A4 | A | Standards of Care in.pdf | 18 | ....In type 1 diabetes, estimates of poor sleep range from 30% to 50% (441), and estimates of moderate to severe... | ....Nighttime lows usually come from how insulin, meals, and your body’s rhythms line up while you’re asleep... | 0.832 | Directly aligned | Directly reflected in response | The retrieved segment discusses nocturnal hypoglycemia and sleep in type 1 diabetes, which directly matches the CA explanation of nighttime lows. |
| 4 | B2 | B | Interpreting CGM data.pdf | 1 | ...for different meal compositions? – If glucose levels do not return to baseline at around 4 hours... | ...Start the meal with vegetables or a salad; a splash of vinegar or lemon can also blunt the rise... | 1.02 | Broadly aligned | Conceptually consistent | The retrieved segment concerns meal composition and post-meal glucose patterns, aligning with the CA dietary strategy. |
| 5 | C4 | C | agp_interpretation.pdf | 4 | ...Insuf/f_icient lunchtime carbohydrate • Unplanned exercise (early morning run) • ... | ...Acarbose is all about timing with the meal. It needs to be taken right with the first bite (or within the first few bites) of ... | 1.16 | Weakly aligned | Background/context only | The retrieved segment relates to meals and glucose patterns, but does not support the acarbose-specific advice. |
| 5 | D3 | D | Diabetes UK Australian Handbook.pdf | 84 | ...glucose reactions’ • ‘feelings of guilt and anxiety when off track with diabetes management’ ... | ... I see most often in your situation are a dawn rise before breakfast and bigger spikes after higher‑carb or restaurant meals… | 0.915 | Weakly aligned | Background/context only | The retrieved segment concerns diabetes-related anxiety, while the displayed CA response mainly addresses glucose pattern interpretation. |
| 6 | E1 | E | CGM-Guided-Glucose-Management.pdf | 1 | ...Every 5% improvement in TIR is clinically beneficial even if not at goal today... | ...ur aim is to keep A1c around 7% or a little lower without hypoglycemia and to spend at least 80–90% of the day between 70 and 180 mg/dL… | 0.766 | Directly aligned | Directly reflected in response | The retrieved segment supports time-in-range goal interpretation, which is directly used in the CA response. |
| 6 | F2 | F | CGM-Guided-Glucose-Management.pdf | 1 | ...Aim for MORE GREEN/ LES S RED in the time in ranges bar. • Individualize goals as appropriate... | ...A CGM measures interstitial fluid, which lags blood by about 5–10 minutes. During fast changes… | 1.12 | Weakly aligned | Background/context only | Both concern CGM interpretation, but the retrieved segment does not support the interstitial lag explanation. |
| 7 | A5 | A | International Consensus on Use of.pdf | 1 | ...When there is a discrepancy between actual HbA1c and the eA1C based on mean glucose, other glucose measurement methods such as fructosamine, glycated albumin, SMBG, and in particular CGM should be used in conjunction with HbA1c measurements... | ...With your long-haul schedule, it is not surprising that your glucose has been bouncing around this week. The main factors appear to be meal timing, meal composition, sleep disruption, missed acarbose doses, and short bursts of physical activity... | 1.02 | Directly aligned | Directly reflected in response | The retrieved segment supports a range of blood glucose fluctuations and is associated with changes in diet and lifestyle, as well as physical activity. |
| 7 | B4 | B | Standards of Care.pdf | 14 | ...Glycemic Goals and Hypoglycemia... diabetesjournals.org/care... dc24s006.pdf... | ...Most low calorie or zero sugar drinks are considered safe for people with diabetes when used in moderation and usually do not raise blood sugar. Replacing sugary soda with a diet version or flavoured sparkling water can reduce spikes and calories... | 1.19 | No clear alignment | No clear contribution | The retrieved text is only a heading or citation fragment and does not address low-calorie or zero-sugar drinks. |
| 8 | C3 | C | International Consensus on Use of.pdf | 5 | ...Because the function of CGM use is to monitor glucose levels with the ultimate goal of improving glycemic control, it makes clinical sense to combine TIR data with other measures. HbA1c level and time in level 2 hypoglycemia is one such combined measure... | ...Based on your data and your last HbA1c of 7.4%, you are close to goal, with most of the problem coming from post-meal glucose rises. The focus should be improving time in range while avoiding hypoglycemia... | 0.996 | Directly aligned | Directly reflected in response | The retrieved segment links TIR, HbA1c, and hypoglycemia, which are explicitly interpreted in the CA response. |
| 8 | D2 | D | Standards of Care in.pdf | 23 | ...Intratrial exposure to vitamin D and new-onset diabetes among adults with prediabetes... Effects of vitamin D supplementation on prevention of type 2 diabetes in patients with prediabetes... | ...When you are under pressure, your body releases adrenaline and cortisol, which tell the liver to put extra sugar into the blood. Stress, sleep loss, and irregular eating can therefore make CGM patterns look worse even when your usual habits have not changed much... | 0.795 | Broadly aligned | Conceptually consistent | The retrieved segment is diabetes-education related but does not directly address stress-related glucose changes. |
| 9 | E3 | E | Standards of Care in.pdf | 7 | ...In the ASCEND trial, supplementation with n-3 fatty acids at the dose of 1 g/day did not lead to cardiovascular benefit in people with diabetes without evidence of CVD. However, REDUCE-IT found that 4 g/day of pure EPA significantly lowered the risk of adverse cardiovascular events... | ...Some people with type 2 diabetes do reduce or even stop certain medicines, but this depends on insulin production, weight, glucose patterns, medication type, and safety. It should be done gradually with clinician supervision rather than stopping medication abruptly... | 1.39 | Weakly aligned | Background/context only | Both concern long-term diabetes care, but the retrieved EPA/cardiovascular content does not support medication withdrawal advice. |
| 9 | F1 | F | Standards of Care.pdf | 14 | ...Training in flexible, intensive insulin management to enable dietary freedom in people with type 1 diabetes... DAFNEplus programme for sustained type 1 diabetes self management... intervention development using the Behaviour Change Wheel... | ...Your CGM is probably working as expected, but there are normal quirks that can make readings look confusing. Interstitial glucose can lag behind blood glucose, compression can cause false lows, and rapid changes may not immediately match fingerstick readings... | 1.41 | No clear alignment | No clear contribution | The retrieved segment concerns insulin education, while the CA response addresses CGM lag and compression lows. |
| 10 | A2 | A | Standards of Care.pdf | 1 | ...The ADA and the American Association for Clinical Chemistry have determined that the correlation in the ADAG trial is strong enough to justify reporting both the A1C result and the estimated average glucose result when a clinician orders the A1C test... | ...Nighttime lows are common with gliclazide. It can keep insulin flowing even without food, so if dinner is early, smaller than usual, or followed by activity, glucose may fall overnight. This should be discussed with a clinician because medication timing or dose may need review... | 0.689 | Directly aligned | Directly reflected in response | The retrieved segment frames glycemic assessment through A1c and estimated average glucose, while the CA applies glycemic interpretation to nocturnal lows. |
| 10 | B6 | B | Standards of Care in.pdf | 7 | ...Periodic testing of vitamin B12 levels should be considered in people taking metformin, particularly in those with anemia or peripheral neuropathy. Routine supplementation with antioxidants, such as vitamins E and C, is not advised due to lack of evidence of efficacy... | ...Gliclazide can make your pancreas release more insulin even when food intake is lower or delayed, which can increase the risk of unexpected lows. This does not mean you are doing something wrong, but it does mean the medication plan may need review... | 1.01 | No clear alignment | No clear contribution | The retrieved segment concerns metformin/B12 and antioxidants, not gliclazide-related hypoglycemia. |
| 11 | C2 | C | Standards of Care in.pdf | 11 | ...When indicated, refer to mental health professionals or other trained health care professionals for further assessment... Facilitating Positive Health Behaviors and Well-being... | ...You are doing a lot right, and it is understandable to feel frustrated when numbers still run high. In type 2 diabetes, glucose can remain elevated because insulin resistance and reduced insulin production change over time. Lifestyle effort still matters, but treatment may also need adjustment... | 0.942 | Broadly aligned | Conceptually consistent | The retrieved segment supports psychosocial and behaviour-change framing, aligning broadly with the CA response on frustration and treatment review. |
| 11 | D4 | D | Diabetes UK Australian Handbook.pdf | 6 | ...Diabetes and emotional health... CBT Cognitive Behavioural Therapy... DDS Diabetes Distress Scale... Diabetes Education and Self-Management for Ongoing and Newly Diagnosed... | ...This is not a failure. Type 2 diabetes can progress over time, and needing additional treatment does not mean you caused the problem. Emotional distress around diabetes is common, and support, education, and practical problem solving can help... | 1.37 | Directly aligned | Directly reflected in response | The retrieved segment directly concerns diabetes distress, CBT, and self-management support, which are reflected in the CA response. |
| 12 | E2 | E | Standards of Care in.pdf | 7 | ...Supplementation with 4 g/day of pure EPA significantly lowered the risk of adverse cardiovascular events. This trial of 8,179 participants, in which over 50% had diabetes, found a 5% absolute reduction in cardiovascular events... People with diabetes should be advised... | ...This goal is realistic. You are close already. A few careful changes can help reach under 6.5% without more lows. The priority is smoothing overnight drops, reducing post-meal spikes, and making small sustainable changes rather than pushing too aggressively... | 1.21 | Weakly aligned | Background/context only | Both relate to long-term diabetes care, but the retrieved EPA content does not support the glycemic goal-setting advice. |
| 12 | F4 | F | Standards of Care.pdf | 14 | ...Training in flexible, intensive insulin management to enable dietary freedom in people with type 1 diabetes... DAFNEplus programme for sustained type 1 diabetes self-management... intervention development using the Behaviour Change Wheel... | ...A gap is usually technical. With Libre, the sensor stores about 8 hours of data. If more than 8 hours pass between scans, older data can be lost. Gaps can also happen from sensor signal issues, phone problems, or temporary disconnection... | 1.54 | No clear alignment | No clear contribution | The retrieved segment concerns insulin management education, not Libre data gaps or connectivity troubleshooting. |

CA: conversational agent; L2: Euclidean distance. Retrieval scores are reported as L2 distances rounded to three significant figures; lower L2 values indicate greater vector similarity between the query and the retrieved segment. Retrieved guideline segments and archived CA responses are shown as truncated excerpts for readability and should not be interpreted as complete source passages or full responses. Relationship categories were assigned by comparing the top-ranked retrieved segment with the archived CA response. Directly aligned indicates that the retrieved segment was explicitly reflected or closely paraphrased in the CA response. Broadly aligned indicates that the retrieved segment was conceptually consistent with the CA response but was not repeated in specific detail. Weakly aligned indicates a limited, indirect, or background-level connection. No clear alignment indicates that the retrieved segment had no meaningful relationship to the CA response or did not appear to contribute substantively to the displayed response.
