# Supplementary material for: Retrieval-Augmented Large Language Model Counseling for Continuous Glucose Monitoring in Diabetes: Source-Masked Multirater Comparative Evaluation
Source: J Med Internet Res. 2026 Jul 31;28:e98519. doi: 10.2196/98519 (PMC13430954; doi:10.2196/98519)
Supplement: Multimedia Appendix 6 [file jmir-v28-e98519-s006.docx]

**Multimedia Appendix 7**

**S 3: Association between response length and quality ratings**

| **Outcome** | **CA β (95% CI)** | **P value** | **Clinician β (95% CI)** | **P value** | **Interaction P value** |
| --- | --- | --- | --- | --- | --- |
| Overall quality | 0.00072 (−0.0006 to 0.0021) | 0.293 | −0.00055 (−0.0016 to 0.0005) | 0.305 | 0.149 |
| Clinical accuracy | 0.00013 (−0.0013 to 0.0016) | 0.858 | 0.00002 (−0.0011 to 0.0012) | 0.979 | 0.902 |
| Guideline adherence | 0.00125 (−0.0002 to 0.0027) | 0.094 | −0.00052 (−0.0017 to 0.0006) | 0.379 | 0.068 |
| Actionability | 0.00027 (−0.0014 to 0.0019) | 0.753 | −0.00038 (−0.0017 to 0.0009) | 0.574 | 0.557 |
| Personalization | 0.00081 (−0.0009 to 0.0025) | 0.345 | −0.00055 (−0.0019 to 0.0008) | 0.413 | 0.211 |
| Clarity | 0.00041 (−0.0012 to 0.0020) | 0.623 | 0.00053 (−0.0007 to 0.0018) | 0.407 | 0.912 |
| Empathy | 0.00107 (−0.0006 to 0.0028) | 0.223 | −0.00235 (−0.0037 to −0.0010) | 0.001 | 0.003 |

Linear mixed-effects models were fitted separately for the overall quality score and each of the 6 quality dimensions. Word count was included as a fixed-effect covariate, along with an interaction term between responder type (CA vs clinician) and word count. Random intercepts were included for unique response ID and rater to account for clustering of ratings (3 raters per response).

Coefficients represent the estimated change in rating score per additional word. “CA β” and “Clinician β” denote the within-group association between word count and rating score for chatbot- and clinician-authored responses, respectively. The interaction P value tests whether the association between word count and rating differs between CA and clinician responses. Two-sided Wald tests were used to assess statistical significance.
