# Supplementary material for: Retrieval-Augmented Large Language Model Counseling for Continuous Glucose Monitoring in Diabetes: Source-Masked Multirater Comparative Evaluation
Source: J Med Internet Res. 2026 Jul 31;28:e98519. doi: 10.2196/98519 (PMC13430954; doi:10.2196/98519)
Supplement: Multimedia Appendix 7 [file jmir-v28-e98519-s007.docx]

**Multimedia Appendix 8**

**S 4:Domain-specific mixed-effects model results comparing CA and clinician responses**

| **Domain** | **Estimated mean difference** | **95% CI** | **P value** |
| --- | --- | --- | --- |
| A | 1.013 | 0.823 to 1.204 | <0.001 |
| B | 0.722 | 0.555 to 0.889 | <0.001 |
| C | 0.912 | 0.695 to 1.129 | <0.001 |
| D | 0.721 | 0.507 to 0.935 | <0.001 |
| E | 0.419 | 0.053 to 0.785 | 0.0248 |
| F | 0.493 | 0.089 to 0.897 | 0.0168 |

Estimated mean differences (CA – Clinician) in overall quality scores between CA-generated and clinician-generated responses within each predefined content domain, derived from linear mixed-effects models with individual ratings as the outcome. Models included responder type as a fixed effect and random intercepts for case and rater. Values represent CA minus clinician mean differences with 95% CIs and two-sided P values.
