# Supplementary material for: Retrieval-Augmented Large Language Model Counseling for Continuous Glucose Monitoring in Diabetes: Source-Masked Multirater Comparative Evaluation
Source: J Med Internet Res. 2026 Jul 31;28:e98519. doi: 10.2196/98519 (PMC13430954; doi:10.2196/98519)
Supplement: Multimedia Appendix 8 [file jmir-v28-e98519-s008.docx]

**Multimedia Appendix 9**

**S 5: Domain- and dimension-specific quality ratings (mean ± SD)**

| **Domain** | **Responder** | **Accuracy** | **Guidelines** | **Actionable** | **Personalized** | **Clarity** | **Empathy** | **Overall Quality** |
| --- | --- | --- | --- | --- | --- | --- | --- | --- |
| **A** | **CA** | 4.35 ± 0.67 | 4.32 ± 0.65 | 4.42 ± 0.67 | 4.31 ± 0.77 | 4.36 ± 0.68 | 4.34 ± 0.67 | 4.35 ± 0.56 |
| **A** | **Clinician** | 3.78 ± 0.97 | 3.69 ± 0.93 | 2.95 ± 1.15 | 3.14 ± 1.19 | 3.53 ± 1.14 | 2.94 ± 1.26 | 3.34 ± 0.94 |
| **B** | **CA** | 4.41 ± 0.64 | 4.36 ± 0.62 | 4.52 ± 0.56 | 4.23 ± 0.67 | 4.51 ± 0.57 | 4.36 ± 0.57 | 4.40 ± 0.46 |
| **B** | **Clinician** | 3.81 ± 0.95 | 3.81 ± 0.94 | 3.69 ± 1.03 | 3.46 ± 1.05 | 3.86 ± 0.96 | 3.41 ± 1.07 | 3.68 ± 0.88 |
| **C** | **CA** | 4.47 ± 0.60 | 4.35 ± 0.77 | 4.38 ± 0.73 | 4.38 ± 0.62 | 4.56 ± 0.58 | 4.38 ± 0.54 | 4.42 ± 0.51 |
| **C** | **Clinician** | 3.72 ± 0.98 | 3.78 ± 0.88 | 3.42 ± 1.12 | 3.35 ± 1.16 | 3.60 ± 1.04 | 3.17 ± 1.23 | 3.50 ± 0.86 |
| **D** | **CA** | 4.38 ± 0.66 | 4.22 ± 0.79 | 4.45 ± 0.69 | 4.26 ± 0.67 | 4.49 ± 0.58 | 4.54 ± 0.60 | 4.39 ± 0.55 |
| **D** | **Clinician** | 3.90 ± 0.92 | 3.82 ± 0.89 | 3.44 ± 1.04 | 3.42 ± 1.06 | 3.78 ± 1.00 | 3.65 ± 1.20 | 3.67 ± 0.86 |
| **E** | **CA** | 4.42 ± 0.73 | 4.25 ± 0.73 | 4.28 ± 0.75 | 4.17 ± 0.77 | 4.31 ± 0.67 | 4.31 ± 0.71 | 4.29 ± 0.63 |
| **E** | **Clinician** | 4.06 ± 0.95 | 4.14 ± 0.96 | 3.79 ± 1.20 | 3.67 ± 1.17 | 3.97 ± 1.00 | 3.58 ± 1.18 | 3.87 ± 0.93 |
| **F** | **CA** | 4.42 ± 1.00 | 4.33 ± 1.01 | 4.35 ± 1.07 | 3.92 ± 1.13 | 4.33 ± 0.96 | 4.14 ± 0.93 | 4.25 ± 0.93 |
| **F** | **Clinician** | 3.97 ± 0.84 | 3.97 ± 0.88 | 3.75 ± 1.02 | 3.56 ± 0.94 | 3.89 ± 1.01 | 3.39 ± 0.99 | 3.75 ± 0.84 |

Domain- and dimension-specific quality ratings (mean ± SD) for CA-generated and clinician-generated responses, presented in paired format for each predefined content domain (A–F). Overall quality represents the arithmetic mean of 6 dimension-specific ratings on a 1–5 scale.
