# Supplementary material for: Retrieval-Augmented Large Language Model Counseling for Continuous Glucose Monitoring in Diabetes: Source-Masked Multirater Comparative Evaluation
Source: J Med Internet Res. 2026 Jul 31;28:e98519. doi: 10.2196/98519 (PMC13430954; doi:10.2196/98519)
Supplement: Multimedia Appendix 9 [file jmir-v28-e98519-s009.docx]

**Multimedia Appendix 10**

**S 6: Rater-level distribution of overall quality scores stratified by perceived source (clinician vs CA)**


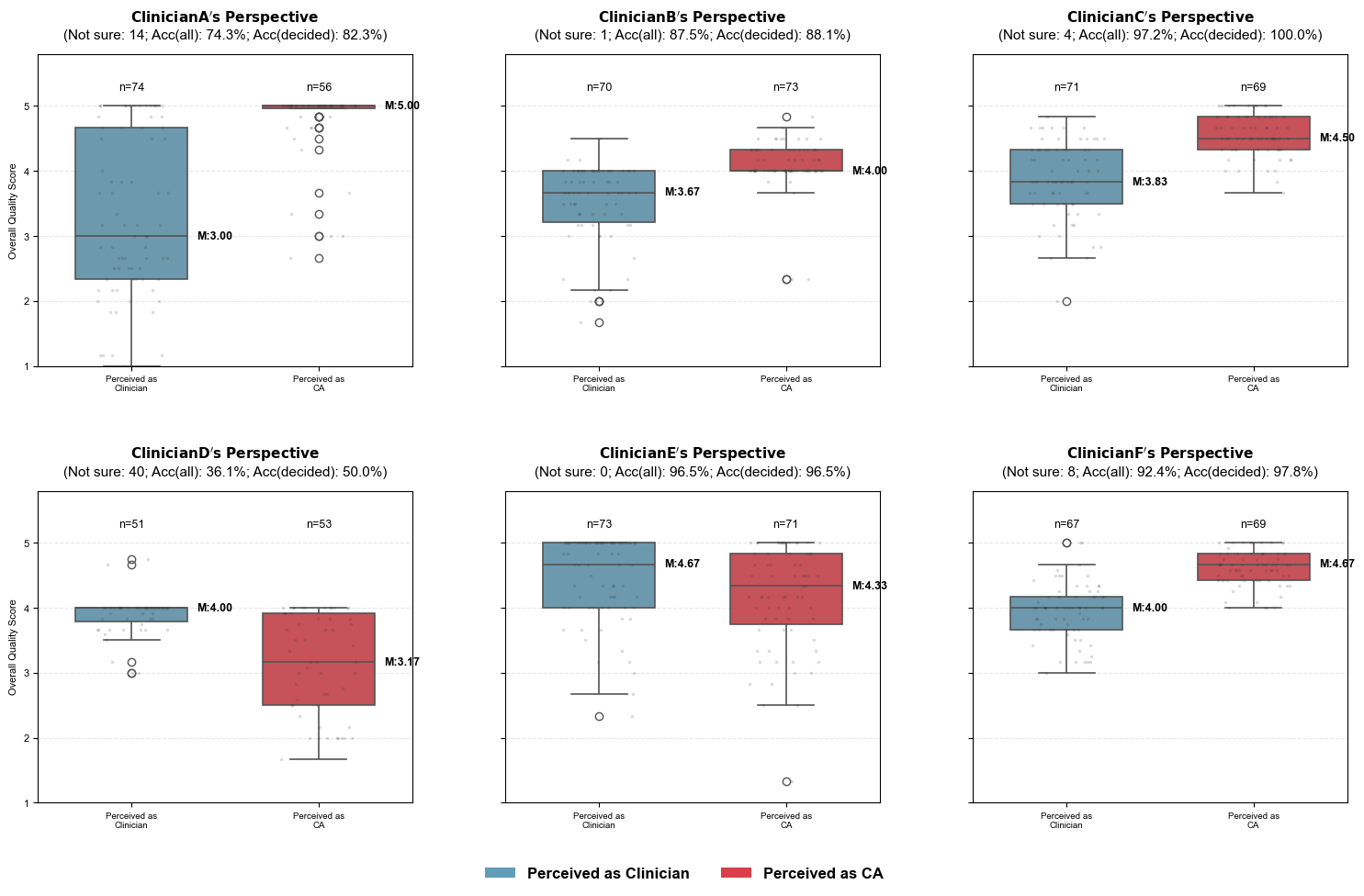


Boxplots show the distribution of overall quality scores assigned by each individual clinician rater (A–F), stratified by the rater’s perceived source of the response (“Perceived as Clinician” vs “Perceived as CA”).

Overall quality was defined as the arithmetic mean of the 6 5-point quality dimensions (clinical accuracy, guideline adherence, actionability, personalization, clarity, and empathy) for each rating instance. In each panel, M denotes the median overall quality score (not the mean). Boxes represent the IQR, horizontal lines indicate medians, and whiskers extend to 1.5×IQR.

The number of responses in each perceived-source category is shown above each box (n). Responses marked as “Not sure” were excluded from the boxplot stratification but are reported separately in the panel subtitle.

Acc (all) denotes identification accuracy calculated across all rated responses, with “Not sure” classifications treated as incorrect.

Acc (decided) refers to identification accuracy calculated only among responses for which the rater made a definitive source judgement (i.e., excluding “Not sure” responses).

These plots illustrate variability in source discrimination performance across raters and allow visual comparison of the quality scores assigned to responses perceived as clinician-generated versus CA-generated. These plots are descriptive and should not be interpreted as isolating a causal effect of perceived source, because perceived source was closely related to true source, response style, and response structure.
