# Supplementary material for: Retrieval-Augmented Large Language Model Counseling for Continuous Glucose Monitoring in Diabetes: Source-Masked Multirater Comparative Evaluation
Source: J Med Internet Res. 2026 Jul 31;28:e98519. doi: 10.2196/98519 (PMC13430954; doi:10.2196/98519)
Supplement: Checklist 1 [file jmir-v28-e98519-s010.docx]

**DECIDE-AI checklist**

| **Section** | **Item number** | **DECIDE-AI theme** | **Adapted reporting item for this study** | **Applicability to this study** | **Reported location** |
| --- | --- | --- | --- | --- | --- |
| Title and abstract | 1 | Title | Identify the study as an early-stage evaluation of an AI-based conversational agent or decision-support and communication-support system, specifying the clinical context and study design. | Applicable | Title |
| Title and abstract | I | Abstract | Provide a structured summary including the intended use of the CA, source-masked vignette-based study design, CGM-informed cases, clinician raters, outcomes, safety assessment, main results, limitations, and conclusions. | Applicable | Abstract |
| Introduction | 2a | Intended use | Describe the targeted clinical problem, the current role and limitations of CGM interpretation in diabetes care, and the intended clinical context. | Applicable | Introduction: Background |
| Introduction | 2b | Intended use | Describe the intended supportive role of the CA for structured CGM interpretation, patient-facing explanation, and preconsultation preparation, while clarifying that it is not intended for autonomous therapeutic decision-making. | Applicable | Introduction: Background; Study Aim; Methods: Safety and Security |
| Introduction | II | Objectives | State the objectives of the simulated, source-masked comparative evaluation. | Applicable | Introduction: Study Aim |
| Methods | III | Research governance | Report ethics considerations, use of publicly available de-identified CGM datasets, synthetic vignettes, clinician expert assessment, and data governance procedures. | Applicable | Methods: Ethical Considerations |
| Methods | 3a | Participants / cases | Describe how CGM-informed cases were selected and constructed, including source datasets, diabetes type distribution, data availability constraints, and use of synthetic vignettes. | Applicable | Methods: CGM Data and Case Vignettes |
| Methods | 3a | Participants / cases | Describe how coherence between synthetic vignettes and underlying CGM traces was assessed, including consistency with diabetes type, treatment modality, CGM metrics, visible glucose patterns, and data completeness. | Applicable | Methods: CGM Data and Case Vignettes |
| Methods | 3b | Users / raters | Describe how clinician contributors and raters were recruited, including clinical background, eligibility criteria, seniority, and role in response generation and rating. | Applicable | Methods: Study Design |
| Methods | 3c | User familiarisation | Describe instructions provided to clinicians before authoring responses and rating anonymized outputs, including response-length guidance, rating criteria, safety flags, and perceived-source labels. | Applicable | Methods: Study Design; Table 1 |
| Methods | 4a | AI system | Describe the CA system, including model identifier, API access timing, absence of fine-tuning, generation parameters, prompt design, multimodal CGM inputs, and RAG configuration. | Applicable | Methods: CA Design |
| Methods | 4a | AI system | Describe retrieval configuration, including corpus sources, chunking, embedding model, FAISS indexing, top-k retrieval, and use of retrieved materials in prompts. | Applicable | Methods: CA Design |
| Methods | 4b | Input data | Describe input data provided to the CA, including CGM traces, CGM summary metrics, synthetic vignettes, contextual information, patient questions, visual CGM materials, and retrieved reference segments. | Applicable | Methods: CGM Data and Case Vignettes; CA Design |
| Methods | 4c | AI outputs | Describe CA-generated free-text outputs, how they were generated, exported, anonymized, labelled, and formatted for source-masked evaluation. | Applicable | Methods: CA Design; Study Design |
| Methods | 5a | Implementation setting | Describe the setting as a simulated, offline, vignette-based, source-masked multi-rater evaluation using CGM-informed cases rather than live clinical deployment. | Applicable | Methods: Study Design |
| Methods | 5b | Clinical workflow | Describe whether the CA was evaluated within a live clinical workflow and whether it affected patient care or clinical decisions. | Not applicable | Not applicable; simulated offline vignette-based evaluation with no live clinical workflow integration |
| Methods | 5c | Human oversight | Describe the role of clinician oversight during development and evaluation, including formative clinical review, clinician-authored comparator responses, source-masked ratings, and safety flag assessment. | Applicable | Methods: CA Design; Safety and Security; Study Design |
| Methods | IV | Outcomes | Specify primary and secondary outcomes, including clinical accuracy, guideline adherence, actionability, personalization, communication clarity, empathy, overall quality, safety flags, perceived source, source-identification accuracy, and retrieval-response alignment. | Applicable | Methods: Study Design; Table 1; Post Hoc Retrieval Audit; Statistical Analysis |
| Methods | 6a | Safety and errors | Define how unsafe, inaccurate, or clinically inappropriate outputs were identified using safety flags and optional qualitative safety comments. | Applicable | Methods: Safety and Security; Study Design; Table 1 |
| Methods | 6b | Safety and errors | Describe how risks to patient safety or potential harm were minimized through de-identified inputs, synthetic vignettes, prompt-based safety constraints, RAG grounding, clinician review, and avoidance of directive medication or dose advice. | Applicable | Methods: Safety and Security |
| Methods | 6c | Failure modes | Describe potential failure modes considered during system design, including CGM misinterpretation, overgeneralized lifestyle advice, excessive reassurance, medication-related specificity, missed high-risk glucose patterns, and mismatch with local prescribing criteria. | Applicable | Methods: Safety and Security |
| Methods | 7 | Human factors | Describe communication-related and human factors dimensions assessed during evaluation, including clarity, empathy, personalization, actionability, and perceived source. | Partially applicable | Methods: Study Design; Table 1 |
| Methods | V | Analysis | Describe statistical methods for primary and secondary outcomes, including ICCs, mixed-effects models, Wilcoxon sensitivity analysis, word-count adjustment, domain-specific analyses, safety summaries, source-identification analysis, and perceived-source analysis. | Applicable | Methods: Statistical Analysis |
| Methods | 8 | Ethics | Describe ethics-related safeguards, including de-identification, use of publicly available datasets, synthetic vignettes, privacy protection, responsible AI use, and absence of direct patient contact. | Applicable | Methods: Ethical Considerations; Safety and Security |
| Methods | VI | Patient and public involvement | State whether patients or public contributors were involved in developing the research question, study design, or evaluation. | Not applicable | Not applicable; no patient or public contributors were involved in this simulated expert-panel evaluation |
| Methods | Additional item | Reporting framework | State that DECIDE-AI was used to guide reporting of this early-stage AI evaluation and specify why it was selected over a prospective trial reporting framework. | Applicable | Methods: Reporting Framework |
| Results | 9a | Participants / cases | Report the number and characteristics of CGM-informed cases, including diabetes type distribution and case-question structure. | Applicable | Results: Rater Characteristics and Inter-Rater Reliability; Methods: CGM Data and Case Vignettes |
| Results | 9b | Users / raters | Report characteristics of clinician raters, including number, gender distribution where available, and clinical experience. | Applicable | Results: Rater Characteristics and Inter-Rater Reliability |
| Results | 10a | Implementation | Report the number of CA-generated and clinician-authored responses evaluated and the total number of rating instances completed. | Applicable | Results: Rater Characteristics and Inter-Rater Reliability |
| Results | 10b | Implementation | Report whether the CA caused changes to clinical workflow. | Not applicable | Not applicable; no live clinical workflow integration |
| Results | VII | Main results | Report main comparative findings for all prespecified quality outcomes, including overall quality and dimension-specific comparisons between CA-generated and clinician-authored responses. | Applicable | Results: Quality Comparisons; Table 3 |
| Results | VIII | Subgroup / exploratory analysis | Report exploratory analyses by domain, response source, rater, response length, and perceived source where performed. | Applicable | Results: Quality Comparisons; Safety and Source Identification; Multimedia Appendices 7-10 |
| Results | 11 | Modifications | Report whether changes were made to the AI system, prompts, retrieval corpus, or output format during formal evaluation. | Applicable | Methods: CA Design; Study Design |
| Results | 12 | Human-computer agreement / source recognizability | Report perceived-source classification, source-identification accuracy, and rater-level heterogeneity in source recognition. | Partially applicable | Results: Safety and Source Identification; Multimedia Appendix 10 |
| Results | Additional item | Retrieval audit | Report retrieval-response alignment, FAISS L2 distance patterns, reviewer agreement, and domain-level retrieval patterns from the post hoc RAG audit. | Applicable | Methods: Post Hoc Retrieval Audit; Results: Post Hoc Retrieval Audit; Multimedia Appendix 5 |
| Results | 13a | Safety and errors | Report significant safety concerns, unsafe recommendations, or output concerns, including safety flag frequencies by response source. | Applicable | Results: Safety and Source Identification |
| Results | 13b | Safety and errors | Report observed or potential risks to patient safety or indirect harm, including medication-policy-sensitive concerns such as GLP-1 eligibility under NHS BMI criteria. | Applicable | Results: Safety and Source Identification; Discussion: Limitations |
| Results | 14a | Human factors | Report communication-related evaluation findings, including clarity, empathy, personalization, actionability, and perceived-source patterns. | Partially applicable | Results: Quality Comparisons; Safety and Source Identification |
| Results | 14b | Human factors | Report user learning curve findings where applicable. | Not applicable | Not applicable; no repeated live use, patient interaction, or workflow learning-curve assessment |
| Discussion | 15 | Support for intended use | Discuss whether findings support the intended role of the CA as an adjunct tool for structured CGM explanation, patient education, and preconsultation preparation. | Applicable | Discussion: Principal Findings; Conclusions |
| Discussion | 16 | Safety and errors | Discuss safety profile, observed concerns, medication-policy-sensitive limitations, local prescribing alignment, and safeguards required before implementation. | Applicable | Discussion: Limitations; Conclusions |
| Discussion | IX | Strengths and limitations | Discuss limitations including simulated vignette-based design, small case set, limited type 2 diabetes representation, rater variability, imperfect source masking, source recognizability, dual clinician role, scaffolded CA versus unassisted clinician responses, and lack of live clinical implementation. | Applicable | Discussion: Limitations |
| Discussion | Additional item | Generalisability | Discuss generalisability to type 1 and type 2 diabetes care, including data-availability constraints and limited representation of broader type 2 diabetes treatment heterogeneity. | Applicable | Discussion: Limitations |
| Discussion | Additional item | RAG interpretation | Discuss the partial and domain-dependent contribution of RAG and caution that guideline adherence ratings should not be interpreted as solely attributable to retrieval grounding. | Applicable | Discussion: Principal Findings; Limitations |
| Discussion | Additional item | Future implementation | Discuss the need for prospective validation in interactive clinical workflows, including patient understanding, consultation efficiency, clinician workload, safety escalation, and local governance. | Applicable | Discussion: Limitations; Conclusions |
| Statements | 17 | Data availability | State whether and how datasets, retrieval corpus, prompts, code, and analysis scripts are available. | Applicable | Data Availability; Code Availability; Multimedia Appendices 1-5 |
| Statements | X | Conflicts of interest | Disclose funding sources, funder roles, commercial involvement, and author conflicts of interest, including coauthor involvement as source-masked raters and/or comparator-response contributors. | Applicable | Conflicts of Interest; Funding Statement |
| Statements | Additional item | Generative AI disclosure | Disclose whether generative AI was used in manuscript preparation and clarify that it was not used to generate data, conduct analyses, assign ratings, adjudicate outcomes, or determine conclusions. | Applicable | Use of Generative AI |
